# Translocation of Antimicrobial Peptides across Model Membranes: The Role of Peptide Chain Length

**DOI:** 10.1021/acs.molpharmaceut.4c00450

**Published:** 2024-07-12

**Authors:** Amanda E. Skog, Nicolò Paracini, Yuri Gerelli, Marie Skepö

**Affiliations:** † Division of Computational Chemistry, Department of Chemistry, Lund University, P.O. Box 124, SE-221 00, Lund, Sweden; ‡ Institut Laue-Langevin, 71 Avenue des Martyrs, 38000 Grenoble, France; ¶ Institute for Complex Systems - National Research Council (ISC−CNR), Piazzale Aldo Moro 2, 00185 Roma, Italy; § Department of Physics, Sapienza University of Rome, Piazzale Aldo Moro 2, 00185 Roma, Italy; ∥ NanoLund, Lund University, Box 118, 22100 Lund, Sweden

**Keywords:** antimicrobial, antifungal, peptide, model membrane, lipid bilayers, cushion formation, solid-supported lipid bilayers, saliva, histatin 5

## Abstract

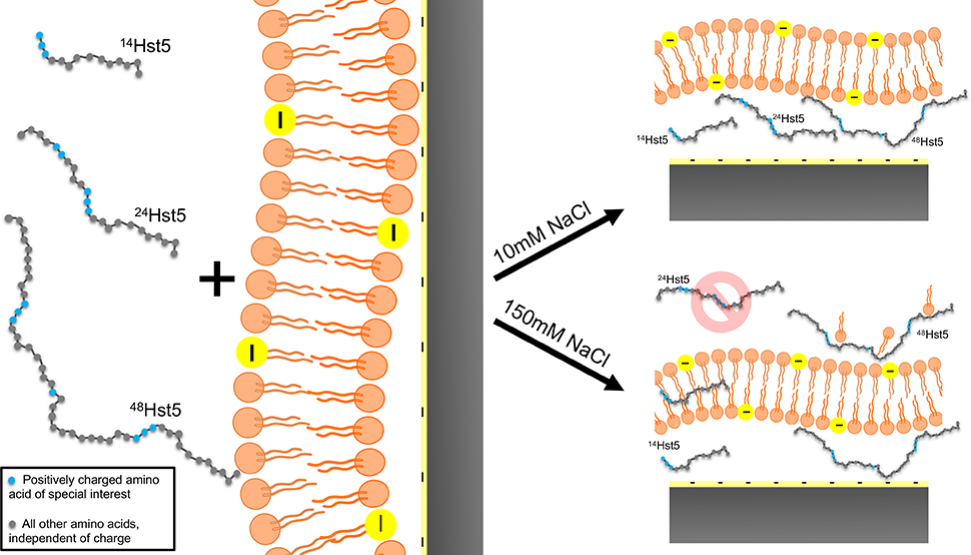

Cushioned lipid bilayers are structures consisting of a lipid bilayer supported on a solid substrate with an intervening layer of soft material. They offer possibilities for studying the behavior and interactions of biological membranes more accurately under physiological conditions. In this work, we continue our studies of cushion formation induced by histatin 5 (^24^Hst5), focusing on the effect of the length of the peptide chain. ^24^Hst5 is a short, positively charged, intrinsically disordered saliva peptide, and here, both a shorter (^14^Hst5) and a longer (^48^Hst5) peptide variant were evaluated. Experimental surface active techniques were combined with coarse-grained Monte Carlo simulations to obtain information about these peptides. Results show that at 10 mM NaCl, both the shorter and the longer peptide variants behave like ^24^Hst5 and a cushion below the bilayer is formed. At 150 mM NaCl, however, no interaction is observed for ^24^Hst5. On the contrary, a cushion is formed both in the case of ^14^Hst5 and ^48^Hst5, and in the latter, an additional thick, diffuse, and highly hydrated layer of peptide and lipid molecules is formed, on top of the bilayer. Similar trends were observed from the simulations, which allowed us to hypothesize that positively charged patches of the amino acids lysine and arginine in all three peptides are essential for them to interact with and translocate over the bilayer. We therefore hypothesize that electrostatic interactions are important for the interaction between the solid-supported lipid bilayers and the peptide depending on the linear charge density through the primary sequence and the positively charged patches in the sequence. The understanding of how, why, and when the cushion is formed opens up the possibility for this system to be used in the research and development of new drugs and pharmaceuticals.

## 1 Introduction

Solid-supported lipid bilayers (SLB) are commonly used to study the structures and interactions of biological membranes. These artificial lipid bilayers, which are formed on solid substrates, offer a platform for studying various biophysical and biochemical processes with high precision and control. By tethering lipid molecules to a solid surface, SLBs provide stability and enable the integration of advanced analytical techniques to explore the intricate dynamics of membrane-associated phenomena. However, they suffer limitations due to the restricted mobility of lipids caused by interactions with the substrate. Cushioned lipid bilayers, are structures consisting of a lipid bilayer supported on a solid substrate with an intervening layer of soft material, and they offer possibilities for studying the behavior and interactions of biological membranes more accurately under physiological conditions. They are particularly relevant in studying reconstituted membrane proteins as the underlying spacer prevents substrate-induced protein degradation and favors protein lateral mobility.^1^ The cushion layer provides mechanical support and flexibility, mimicking the dynamic properties of cellular environments more closely than traditional SLBs. The cushion formation is usually achieved by assembling the SLB directly onto a polymer-functionalized surface, which can affect adsorption processes such as vesicle fusion required for SLB formation. Therefore, understanding how cushion formation can be induced on an assembled SLB can add a useful tool to creating cushioned membranes, which holds significant implications across diverse fields, ranging from fundamental biophysical research to developing novel biomaterials and drug delivery systems.

Histatin 5, referred to as ^24^Hst5, is a histidine-rich, intrinsically disordered, and multifunctional peptide found in saliva. It acts as a first line of defense against oral candidiasis caused by *Candida albicans*.^2−7^ We have previously shown that when added to a SLB, ^24^Hst5 is capable of intercalating in between the model lipid membrane and the underlying substrate, forming a peptide cushion. The cushion formation, which did not alter the integrity of the membrane, was shown to depend on several factors, such as the ionic strength of the buffer, if a negatively or positively charged solid substrate was utilized, the charge density of the bilayer,^8^ and the number of histidines in the peptide sequence.^9^ This behavior of the peptide is in line with the mode of action observed for its antimicrobial effect, where ^24^Hst5 has been shown to translocate across the cell membrane and accumulate intracellularly in the mitochondrion.^10−12^ It was shown crucial that there is a membrane potential on the mitochondrion,^13^ that is, there needs to be a negative charge inside the plasma membrane for the peptide to be active against the target microbe.

**Figure 1:**
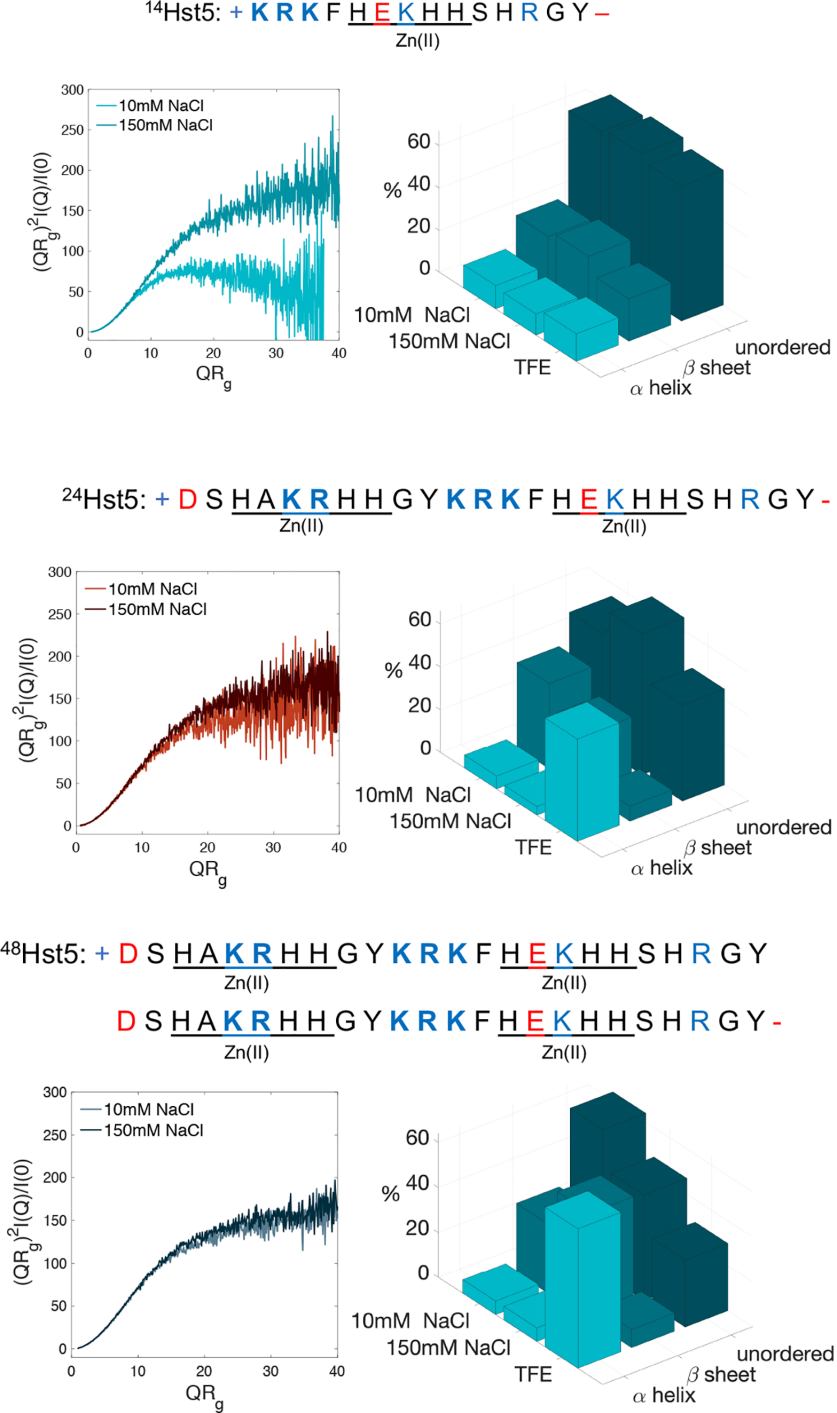
Peptide primary sequences, normalized Kratky plots at 10 and 150 mM NaCl concentration, and circular dichroism (CD) fits obtained as an average from SELCON3/SELCON2 and BeStSel^29,30^ in aqueous buffer, 10 and 150 mM NaCl, as well as TFE of ^14^Hst5 (top), ^24^Hst5 (middle), and ^48^Hst5 (bottom). The charged patches and positively and negatively charged amino acids are in blue and red, respectively. The zinc motifs are underlined.

In this study, the aim is to further investigate the mechanism allowing the peptide to translocate across the bilayer without disrupting the model membrane. Here, both a shorter variant, ^14^Hst5,^4,14−20^ corresponding to the last 14 amino acids of ^24^Hst5, and a longer variant, ^48^Hst5,^21^ being the tandem repeat of ^24^Hst5 are investigated and compared to ^24^Hst5. The sequence of these peptides are presented in Figure 1. The histidine ratio is kept constant in both variants at 29%, which is the same ratio found in ^24^Hst5. Regarding the charges and charge distribution of these peptides, the ratio of charged amino acids are the same in ^48^Hst5 as ^24^Hst5, as it is the tandem-repeat of the peptide. For ^14^Hst5, the linear charge density is actually slightly higher compared to ^24^Hst5 since the sequence contains only two fewer positively charged residues and one less negatively charged amino acid. Still, the overall length is reduced by ten residues.

These peptides all contain positively charged patches of the basic amino acids lysine (K) and arginine (R), such as KR and KRK repeats, indicated in bold in Figure 1, which were recognized to be similar to those found in nuclear-localizing sequences (NLS), as well as sequences of cell-penetrating peptides (CPP).^22,23^ NLSs are responsible for directly importing proteins into the nucleus and are typically short, consisting of basic amino acids. They are also expected to contain α-helix disruptive amino acids such as proline.^24^ CPPs are short peptides of 5–30 amino acids with a net positive charge that can penetrate biological membranes and deliver cargo into the cell. The mechanism of action for these peptides is still not fully known, but it has been established that all proposed routes start with the interaction of the bilayer.^25^^24^Hst5 has previously been suggested to be a CPP^13,26^ since it targets the mitochondria to kill the targeted microbe. The ability to translocate the cell membrane opens up the possibility of the peptide to be used to carry cargo of pharmaceutical molecules into the cell.

We hypothesize that the charged patches of K and R in the peptide sequence are important for the ability of the peptide to translocate across the bilayer. The objectives of this study are two-fold, namely, (i) to investigate how the length of the peptide affects the interaction with the model membrane, mainly the penetration depth, which in turn can be connected to its ability to possibly carry cargo across the cell membrane, and (ii) to investigate the importance of the charged patches for the ability to adsorb to the top of the bilayer, a requirement for further penetration into the bilayer. Objective (i) is investigated in high and low salt concentrations, whereas objective (ii) is performed only in low salt concentrations. However, the results are compared with those of the high salt concentration.

This study combines experimental techniques, such as neutron reflectometry (NR), quartz-crystal microbalance with dissipation monitoring (QCM-D), small-angle X-ray scattering (SAXS), and circular dichroism (CD), with coarse-grained Monte Carlo (MC) simulations to obtain information about the system. Results show that at low NaCl concentration, 10 mM, both the shorter and the longer peptide variants behave like ^24^Hst5 and that a cushion below the bilayer is formed. At high NaCl concentration, 150 mM, however, no interaction is observed for ^24^Hst5. At the same time, the shorter peptide displays similar behavior in low salt concentration with cushion formation and some additional peptide within the bilayer. In the case of ^48^Hst5, a cushion below the bilayer is again observed; however, a thick and diffuse adsorbed layer of peptide and lipid molecules is formed on top of the bilayer.

## 2 Experimental Section

### 2.1 Peptide Solutions

^14^Hst5, ^24^Hst5, and ^48^Hst5, were purchased from TAG Copenhagen A/S, Denmark, with a purities of 95, 99, and 95%, respectively, determined by high-performance liquid chromatography (HPLC). Before use, the peptides were further purified by dialysis using a 100 to 500 Da MWCO Biotech Cellulose Ester (CE) Dialysis Membrane Tubing (SpectrumLabs, Piraeus, Greece) against Milli-Q water at 6–9 °C and lyophilized. Finally, the peptide powder was dissolved in the correct solution for each experiment, as described in the corresponding section.

### 2.2 Vesicle Preparation and Vesicle Fusion Protocol

Lyophilized 1-palmitoyl-2-oleoyl-*sn*-glycero-3-phosphocholine (POPC) and 1-palmitoyl-2-oleoyl-*sn*-glycero-3-phospho-l-serine (POPS) as well as their partially deuterated homologues d_31_POPC (1-palmitoyl-*d*_31_-2-oleoyl-*sn*-glycero-3-phosphocholine) and d_31_POPS (1-palmitoyl-*d*_31_-2-oleoyl-*sn*-glycero-3-phospho-l-serine) were purchased from Avanti Polar Lipids (Alabaster, USA). Stock solutions were prepared in a 3:7 methanol:chloroform mixture using the lipid molar ratio POPC:POPS 9:1, referred to as PC:PS 9:1 in the text. The use of partially deuterated lipids is also indicated in the acronyms (usually as d_31_).

The methanol:chloroform mixture was evaporated under nitrogen flow to form a lipid film, and any remaining solvent was evaporated under reduced pressure, 0.8 bar. The lipid films were hydrated in 500 mM NaCl, and 20 mM TRIS buffer at pH 7.4. Small unilamellar vesicles, SUVs, were obtained by tip sonication (Bandelin Sonopuls) for a total of 30 min with 30% maximum amplitude, by pulsing with 2 s ON and 3 s OFF.

To obtain SLBs, the vesicle fusion^27,28^ protocol optimized and described previously by us^8^ was utilized. In brief, the injection of vesicles was performed in a buffer containing 500 mM NaCl buffer, and the vesicles were left to incubate for 60 min, followed by a rinsing step with Milli-Q-H_2_O to induce osmotic shock. This resulted in reproducible, high-quality PC:PS 9:1 bilayers.

#### 2.2.1 Circular Dichrosim

The peptides were dissolved in either aqueous buffer, 20 mM phosphate buffer at pH 7.4, complemented with either 10 or 150 mM NaF or 2,2,2-trifluoroethanol (TFE) to a peptide concentration of 0.1 mg mL^–1^ for all peptides. Far-UV CD measurements were performed on a JASCO J-715 spectropolarimeter with a photomultiplier tube detector. Spectra were recorded every 1.0 nm in the range of 185–260 nm. The temperature was kept at 20 °C, and measurements started after 5 min of equilibration. Subtraction of reference spectra containing only buffer or TFE was performed on all spectra. The measured ellipticity is reported as Delta Epsilon (cm^–1^ M^–1^) according to
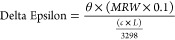
1where θ is the measured ellipticity (mdeg), *MRW* is the mean residue molecular weight, *c* is the peptide concentration (g mL^–1^), and *L* is the optical path length of the cell (cm).

The obtained CD spectra were analyzed and fitted using two different methods: BeStSel,^29,30^ see http://bestsel.elte.hu/index.php, as well as SELCON3. The code for this function was initially made in Matlab in 2005 by the research group of Wallace (Birkbeck College, London, UK). The MatLab code was updated in 2006–2007 to allow plotting and calculating the mean refitted spectrum to the query protein by Hoffmann (Aarhus University, Denmark). This code was adapted to Python by Hoffmann (Aarhus University, Denmark) in 2021, with the SP175 data set. These different methods gave information on the secondary structure elements present.

#### 2.2.2 Small Angle X-ray Scattering

SAXS measurements were performed at the European Synchrotron Radiation Facility (ESRF; Grenoble, France) using the BioSAXS beamline BM29^31^ to gain information about the structure and possible aggregation behavior. The peptide stock solutions were diluted to desired concentrations in series of approximately 0.5, 1, 2, and 5 mg mL^–1^, and the diluted samples were centrifuged at 14 000 rpm at room temperature for at least 30 min to remove potential large aggregates and/or impurities. The final concentration was determined using a Nanodrop 1000 instrument at 280 nm wavelength, the analyte parameters, molecular weight (*M_m_*) of 1847, 3036, and 6055 Da, and extinction coefficient of 1490, 2580, and 5960 cm^–1^ M^–1^ were used, for ^14^Hst5, ^24^Hst5, and ^48^Hst5, respectively. The SAXS data was obtained using an energy of 12.5 keV and a sample-to-detector distance of 2.867 m resulting to a *Q* range of 0.0044–0.52 Å^–1^. *Q* is defined according to
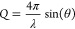
2where λ is the X-ray wavelength, and θ is the scattering angle.

Samples were loaded into a flow-through quartz capillary using an autosampler robot (Arinax). Ten consecutive frames with an exposure time of 1 s each were recorded at 20 °C under flow to reduce radiation damage. The SAXS spectrum of the background, represented by the dialysis buffer, was measured before and after each sample acquisition, using the same exposure time as for the sample. The measurements were performed in replicates for the lowest concentrations, and final averages were determined in the data process. The forward scattering at *Q* = 0, *I*(0), was converted to absolute scale by measuring water scattering. The SAXS integration and initial processing used the BM29 automated pipeline.^32^ For the analysis of the data, the software Primus from the ATSAS package^33^ was utilized. The radius of gyration (*R*_*g*_) was determined for each sample by Gunier analysis in a *Q* range where the relation *Q* × *R*_*g*_ ≤ 1.1 held.

### 2.3 Quartz-Crystal Microbalance with Dissipation Monitoring

QCM-D measurements were performed on an E4 apparatus (Biolin Scientific, Sweden) with four thermally controlled flow modules. All experiments were conducted on SiO_2_-coated AT-cut 5 MHz quartz sensors (Biolin Scientific, Sweden). Before use, the sensors were cleaned and treated as described previously by us.^8^ The cleaned sensors were enclosed in the dry flow modules. Before measurements, the flow modules were filled with buffer, 500 mM NaCl, 20 mM TRIS, pH 7.4, using a peristaltic pump (Ismatec IPC-N 4, Switzerland). All solutions were injected at a constant flow rate of 0.150 mL min^–1^ during the measurement, and data were collected continuously. SLBs were formed according to the vesicle fusion protocol described above. Once the bilayer was formed and rinsed to remove any unbound lipid aggregate, the frequency and dissipation values were set to zero. Peptide-containing solutions, 1 mg mL^–1^ in buffer, 10 mM/150 mM NaCl, 20 mM TRIS, pH 7.4, were injected in the cells and incubated for ∼60 min, during which the pump was off. The samples were then rinsed with buffer for another 60 min. The temperature was kept constant at 20 °C during the entirety of the measurement. QCM-D data were analyzed by evaluating the trends of the normalized frequency shifts, , *n* being the overtone number, and Δ*F*_*n*_ is the frequency response at the *n*th overtone, and of the dissipation factors, Δ*D*_*n*_. In the case of rigid thin films, the Sauerbrey equation can be used to evaluate changes in adsorbed mass per unit area, Δ*m*, as^34,35^
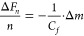
3where *C*_*f*_ is the mass sensitivity constant, *C*_*f*_ = 17.7 ng cm^–2^ Hz^–1^, for an AT-cut quartz crystal with 5 MHz fundamental frequency. When eq 3 holds, the adsorbed mass can also be converted to an equivalent thickness
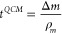
4where ρ_*m*_ is the mass density of the peptide obtained from the ratio between the molecular mass, *M*_*m*_, and the molecular volume, *M*_*v*_, of the peptide species under investigation. Values utilized in the present work are *M*_*v*_: 2235, 3674, and 7327 Å^3^ for ^14^Hst5, ^24^Hst5, and ^48^Hst5 respectively. Since all QCM-D experiments were performed in at least triplicate, the average Δ*F*_*n*_/*n* value for each overtone was calculated using all measurements. This information is reported in all QCM-D graphs in the present manuscript and in the Supporting Information. Once stabilized, the QCM-D traces are usually characterized by small fluctuations around the average value. These fluctuations were used to determine the absolute uncertainty of the Δ*F*_*n*_/*n* values in terms of one standard deviation. Then, eqs 3 and 4 were applied to the data of samples showing a rigid film behavior and, in general, to those data sets showing a less than 10% deviation of the individual overtone Δ*F*_*n*_/*n* values from the average. Error propagation rules were applied to calculate the absolute uncertainty on Δ*m* and, subsequently, on *t*^*QCM*^.

### 2.4 Neutron Reflectometry

NR experiments were performed using silicon single crystals as solid substrates, 8 × 5 × 1.5 cm^3^, cut along the 111 plane, polished to <5 Å root-mean-square (RMS) roughness (Sil’tronix ST, Archamps, France). The cleaning procedure was the same as for QCM-D experiments, except that the substrates were exposed to air plasma for 2 min. After cleaning, the substrates were assembled into water-filled solid/liquid cells. The cells were composed of a water reservoir equipped with inlet and outlet valves, allowing the exchange of aqueous solution and injection of peptide solution. This controlled solution exchange is also required to apply the contrast variation method^36^ and was performed using an HPLC pump.

NR measurements were performed on FIGARO,^37^ the time-of-flight horizontal-surface reflectometer at Institut Laue Langevin (ILL; Grenoble, France). During the experiments, the instrument was configured to operate with incident wavelengths ranging from 2 Å to 20 Å and two angles of incidence, namely, 0.8 and 3.0°, resulting in a *Q*_*z*_ range of 0.0045–0.3 Å^–1^. To exploit the contrast variation method, measurements were performed mixing, at different ratios, D- and H-buffers, D_2_O- and H_2_O-based, respectively: 100% D-buffer, 100% H-buffer, a 38:62 D/H-buffer mixture referred to as silicon-matched buffer (SiMB) with a scattering length density (SLD) value matching that of crystalline silicon and a 66:34 D/H-buffer mixture denoted 4-M buffer, with an SLD value of 4 × 10^–6^ Å^–2^. Pristine SLBs were measured in two contrasts given the simpler structure while SLB+peptide were measured in two to four contrasts depending on the difference observed in the data upon addition of the peptides. Raw data were converted to reflectivity curves using the COSMOS routine.^38^ The silicon substrates were characterized in both 100% D-buffer and 100% H-buffer before injection of vesicles at a concentration of 0.2 mg mL^–1^. After an incubation of 1 h and subsequent rinsing steps, the peptides were injected at a concentration of 1 mg mL^–1^.

Information about the samples was derived by fitting the reflectivity data sets measured under multiple contrasts using a common slab model and the software application Aurore.^39^ The model consisted of a series of layers, each described in terms of SLD, layer thickness *t*, buffer volume fraction *f*_*w*_, and interfacial roughness σ. The model for the bare substrate consisted of an infinite layer with the SLD of the crystalline silicon, an oxide layer, and an infinite bulk aqueous layer. Upon SLB formation, an additional five layers were included to describe the water gap between the solid substrate and the bilayer, followed by the headgroups and tail region of the inner leaflet facing the solid substrate, as well as the tails and head regions of the leaflet in the proximity of the aqueous bulk phase. A schematic representation of this model can be found in ref.^40^ Different scenarios were evaluated for the data obtained after peptide incubation to determine the most suitable model. It was found unnecessary to increase the number of layers in the model; indeed, data could be analyzed simply by allowing changes in the thickness of the water gap between the SLB and substrate and the SLD values of the existing layers to account for the presence of peptide molecules. This holds for all cases except ^48^Hst5 in 150 mM NaCl buffer, in which an additional layer had to be added on top of the bilayer to fit the data. The total SLD of a layer composed of *N* chemical species can be calculated as
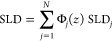
5where Φ_*j*_(*z*) (∑_*j*=1_^*N*^Φ_*j*_(*z*) ≡ 1) is the volume fraction profile, and SLD_*j*_ is the SLD assigned to the *j*th layer in the model. The presence of hydration water was directly accounted for in the model using an additional volume fraction parameter, *f*_*w*_, as described in ref (39). The effect of the exchange of labile protons in the POPS headgroup had to be taken into account to analyze NR data obtained in different H/D-buffer mixtures properly. Proton–deuterium exchange in POPS headgroups was explicitly included in the modeling by modifying the scattering length of the PS headgroup using the lipid plugin provided by the Aurore software. For the peptides, as they were prepared and injected in H_2_O, contrast variation was applied by flushing the cells after the incubation. An average SLD value for the peptides of 2.4 × 10^–6^ Å^–2^ to analyze NR data measured in all contrast conditions. A detailed justification for the use of this value is presented.^8^ The values of the structural parameters and their associated uncertainties were obtained using the built-in routines for nonlinear minimization provided in the MINUIT package and included in the Aurore software application.^39^

A key parameter in the current study is the thickness of the gap formed upon the interaction of the peptides with the SLBs. To compare the results obtained from NR to those obtained from the analysis of QCM-D data, the absolute thickness determined from NR was converted to the equivalent thickness *D*_*j*_ = Φ_*j*_ × *t*_*j*_ which represents the thickness of a layer entirely composed of the *j*th molecular species.^41^ In the case of the gap layer, this quantity is indicated as *D*_*gap*_.

## 3 Computational Section

### 3.1 The Coarse-Grained Model

In the simulations performed for this study a coarse-grained model of the peptides have been utilized, where, instead of considering all the atoms present in the peptide, the amino acids are represented by hard spheres. Both termini are defined as additional residues to account for the extra charge they give rise to. The beads can be negatively charged, positively charged, or uncharged depending on the amino acid sequence at pH 7.4. The simulation includes either none, one, or two surfaces representing the head groups of a lipid bilayer or a solid silica surface. Both surfaces are represented by hard spheres distributed on a primitive cubic lattice, where the particles are frozen in their initial position; hence an approximation of the real system. The surface representing the head groups is built up of 156 particles distributed with 64 Å^2^ between the particles, and each particle was given a charge of −0.5*e*. 990 particles comprise the silica surface, where each particle has a charge of −0.05*e*. The counterions are treated explicitly, whereas the salt is treated implicitly using the Debye–Hückel theory, in which the solvent is treated as a dielectric continuum. Each particle in the simulation has a radius of 2 Å. All nonbonded interactions are assumed to be pairwise additive. There are four contributions to the total energy, three nonbonded and one bonded. Each contribution is described in detail below:
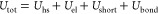
6

The hard sphere potential, *U*_hs_, is given by
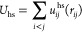
7in which all the particles in the system are included. *r*_*ij*_ is the center-to-center distance between particle *i* and particle *j*. The hard sphere potential between two particles in the model is given by
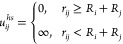
where the radius of particle *i* is given by *R*_*i*_.

The electrostatic potential, *U*_el_, is given by an extended Debye–Hückel potential:
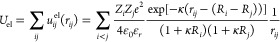
8where all the particles in the system are included in the sum. *Z*_*i*_ is the valency of particle *i*, *e* is the elementary charge, ε_0_ is the permittivity of vacuum, ε_*r*_ is the dielectric permittivity for water, and κ denotes the inverse Debye screening length.

A short-ranged attractive interaction contributing to the total potential energy corresponds to the van der Waals interaction. It is given by
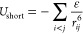
9where ε determines the interaction strength, the attraction is defined to act between all beads in the chain, and in this study, an attractive potential of 0.6 kT at closest contact was used.^42^

The bond energy, *U*_bond_, only applies to the bonded beads in the chain and is given by
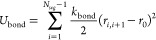
10where *N*_*seg*_ is the number of segments, referred to as beads, in the chain, *r*_*i*,*i*+1_ is the center-to-center distance between two connected beads with the equilibrium distance *r*_0_ = 4.1 Å, and *k*_bond_ = 0.4 N m^–1^ is the force constant.^44^

### 3.2 Monte Carlo Simulation Method

The equilibrium properties of the peptide were obtained by applying MC simulations in the canonical (*NVT*) ensemble, meaning a constant volume, number of beads, and temperature, *T* = 293 K, utilizing the Metropolis algorithm. The peptide chain was enclosed in a rectangular box of variable volume, varying the *z*-length of the box from 20 Å to 100 Å, depending on the system of interest. Periodic boundary conditions were applied in *x*- and *y*-directions. The long-ranged Coulomb interactions were truncated using the minimum image convention. Four types of displacements were allowed: translational displacement of a single bead, pivot rotation, translation of the entire chain, and slithering move. The probability of the different trial moves was weighted to enable single particle moves to occur more often than the other three. In all cases, one surface was placed at *z* = 0 and the other at *z* = 1, meaning the other end of the box. No movements were allowed for either surface or surface particles. The peptide and the counterions were randomly distributed in the box, and an equilibrium simulation of 1 × 10^5^ trial moves per bead was performed. In contrast, the proceeding production run comprised 1 × 10^6^ passes divided into ten subdivisions. The simulations used the integrated Monte Carlo/molecular dynamics/Brownian dynamics simulation package Molsim.^43^ Analysis of the end-to-end distance (*R*_*ee*_) and the radius of gyration (*R*_*g*_) was obtained for the peptide. In the analysis regarding the adsorption probability, a bead was considered adsorbed to the surface if it was closer than 9 Å, which effectively corresponds to 5 Å, due to the radius of the bead and the particle being 4 Å in total. Adsorption probability is defined as the number of passes in which the bead is within adsorption distance from the surface, divided by the total number of passes in the simulation. For all simulated quantities, the reported uncertainty is one standard deviation of the mean. It is estimated from the deviation among the means of the subdivisions of the total number of MC passes, according to
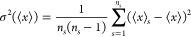
11where ⟨*x*⟩_*s*_ is the average of quantity *x* from one subdivision, ⟨*x*⟩ is the average of *x* from the total simulation, and *n*_*s*_ is the number of subdivisions.

## 4 Results and Discussion

### 4.1 Solution Behavior of the Studied Peptides

To study the length effect of the peptides on the cushion formation, three different peptides have been used: (i) ^24^Hst5, (ii) ^14^Hst5, and (iii) ^48^Hst5 at low, 10 mM, and high, 150 mM, NaCl concentration, as well as in TFE. The latter was used to understand the maximum extent of secondary structure conformation the peptides can obtain. ^24^Hst5 was our reference system. The peptides are characterized experimentally in bulk using CD and SAXS. As previously shown, ^24^Hst5 behaves as a monomeric, unordered chain with a minor secondary structure in an aqueous solution.^4,9,44−47^ The analysis indicates only a slight difference between the two salt concentrations, where the predicted amount of β-sheets is slightly smaller in the latter, shown in Figure 1. However, when the peptide is dissolved in TFE, it becomes significantly more ordered. The predicted amount of α-helices is highly increased, as previously shown.^45,48^ Normalized Kratky plots for both salt concentrations were obtained from SAXS and are shown in Figure 1. No significant salt effects are observed, neither for the shape in the Kratky plot nor in the intraparticle distance distribution function, *P*(*r*). Upon addition of salt and screening of the electrostatic interactions, there is an extension of the maximum distance of 6.5 Å, which corresponds to approximately 12%. Hence, in correspondence with previous measurements, the overall picture is that ^24^Hst5 behaves as an unordered peptide in solution.^9,44^

As anticipated, from a visual inspection of the CD spectra (see Figure S1), ^14^Hst5 and ^48^Hst5 resemble the same features as ^24^Hst5. However, from the CD fits, it is shown that the predicted β-sheet content is lower in ^14^Hst5 and principally unaffected by changing the salt concentration of the buffer, as shown in Figure 1. On the contrary, ^48^Hst5 shows the opposite behavior to ^24^Hst5 where it is predicted to contain more β-sheets in 150 mM compared to 10 mM NaCl. The largest difference between the two variants and ^24^Hst5 is observed in TFE, where the α-helix formation is completely lacking in ^14^Hst5, indicated both by the lack of shifts at λ = 222 nm, as well as λ = 209 nm (see Figure S1, as well by the fits shown in Figure 1). The weaker shift obtained from the helical structure for ^14^Hst5 was also observed in ref (4) and attributed to the shorter sequence length. However, in that study, the helical content in TFE was greater than we observed here. This indicates that the N-terminal, which is removed in ^14^Hst5, compared to ^24^Hst5, could be important for the α-helix formation. The helical content in ^48^Hst5 was observed to be greater than in ^24^Hst5, which aligns with the fact that helix formation depends on the sequence length.

The conformational properties of ^48^Hst5 obtained from SAXS show similar salt dependence as ^24^Hst5. From *P*(*r*) (see Figure S8), it is also clear that the average size is the same in both salt concentrations; however, there is a more significant difference between the maximal length of the peptide, where it is almost 10 Å longer in 150 mM NaCl buffer, hence an increased extension of approximately 16%. For ^14^Hst5, the normalized Kratky plot indicates a transition from unordered to globular when the electrostatic interactions are screened. This is also in line with *P*(*r*), where an average extended conformation is more common in 10 mM NaCl.

### 4.2 Interaction Between the Peptides and Supported Lipid Bilayers

#### 4.2.1 The Effect of Chain Length at Low Salt Concentration

**Figure 2:**
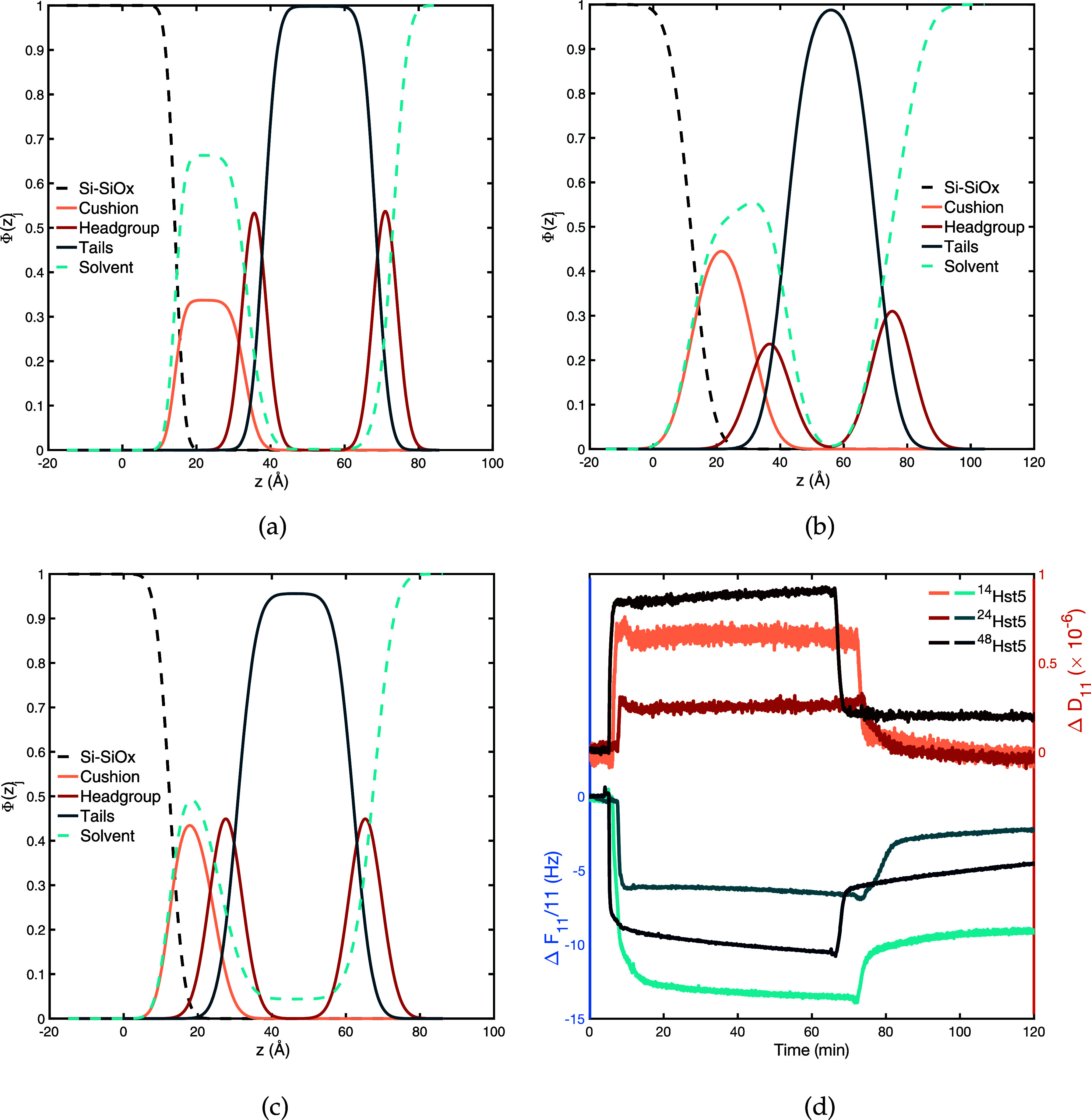
Volume fraction profile components, ϕ_*j*_(*z*), obtained from the analysis of the neutron reflectometry (NR) data measured after the interaction and rinsing, of (a) ^14^Hst5, (b) ^24^Hst5, and (c) ^48^Hst5 at 10 mM NaCl with a PC:PS 9:1 supported lipid bilayer (SLB). The width of the SLB regions differs between the samples as it reflects the roughness of the solid substrate used; the lower the roughness, the more defined the structural features. For clarity, the volume fration profile (VFP) of the crystalline silicon and its oxide are shown together. (d) Frequency shifts (Δ*F*_*n*_/*n*) and dissipation factors (Δ*D*) obtained for the 11th overtone from quartz-crystal microbalance with dissipation monitoring (QCM-D) experiments of ^14^Hst5, ^24^Hst5 and ^48^Hst5 at 10 mM NaCl.

To investigate how the interaction between the peptide and a negatively charged lipid bilayer changes as a function of peptide length, SLD and volume fraction profiles (VFPs or ϕ(*z*)) were derived from the modeling of NR data and adsorbed amounts and adsorption behaviors were obtained from QCM-D data. When ^24^Hst5 was added on the surface of a negatively charged bilayer deposited on top of a negatively charged silica surface in 10 mM NaCl buffer, QCM-D data showed fast adsorption which stabilizes at Δ*F*_11_/11 = −6.5 Hz during incubation, corresponding to an adsorbed mass of 115 ng cm^–2^, according to eq 3. The QCM-D data obtained at low salt concentration is shown in Figure 2d, where only the 11th overtone is presented to make the figure clearer to the reader (the full set of overtones can be found in SI). During incubation, the dissipation increases as the frequency decreases and stabilizes at Δ*D*_11_ = 0.2 × 10^–6^. This indicates that the adsorbed layer can still be considered rigid and compact, further supported by the overlapping harmonics in all measured cells, shown in Figure S16. After incubation, the system was rinsed with buffer to remove any loosely bound or unbound peptide. During this step, the frequency increased again. It stabilized at Δ*F*_11_/11 = −2.3 Hz, with a simultaneous decrease of the dissipation back to zero, indicative of a transition from a less to a more compact film. The adsorbed mass after rinsing is determined to 41 ng cm^–2^. Furthermore, it is impossible to capture a time dependence during incubation or rinsing, indicating that the adsorption and removal of unbound molecules are very fast compared to the experimental time scale. From the obtained masses, eq 4 could be used to determine the thickness of the adsorbed peptide layer, which resulted in 3.0 Å. In contrast to QCM-D, where we mainly get information about the adsorbed amount of peptide, from the analysis of NR data, we obtained information regarding the position of the peptide with respect to the substrate and to the SLB after rinsing. The VFP of ^24^Hst5, previously reported in refs (8) and (9), is presented in Figure 2b and shows that a peptide cushion is formed.

The formed peptide cushion is highly hydrated, as illustrated by the significant volume occupied by the solvent; see the light blue dashed line in Figure 2(a–c). The thickness of the formed cushion is very similar between the ^24^Hst5, 20 ± 1 Å, and the shorter ^14^Hst5, 19 ± 1 Å. The longer variant, ^48^Hst5, does however give rise to a slightly thinner cushion of 12 ± 2 Å. Despite these differences in absolute thickness, the adsorbed amount of peptide, quantified by *D*_*gap*_ (9 ± 1, 6 ± 1, and 6 ± 1 Å, respectively) does not differ significantly among the three samples indicating a different molecular organization of the peptide chains within the gap region.

Both ^14^Hst5 and ^48^Hst5 display a more significant frequency shift, as well as a more considerable increase in dissipation when injected into the SLB compared to ^24^Hst5, shown in Figure 2d.

^14^Hst5 displays a similar behavior as ^24^Hst5 where no time dependence is evident from the obtained data, and the adsorption and removal of unbound molecules can therefore be considered faster than the experimental time scale. Upon injection of peptides, the frequency drops and stabilizes at Δ*F*_11_/11 = −13 Hz, and after rinsing at Δ*F*_11_/11 = −9 Hz, corresponding to a thickness of *t*^*QCM*^ = 11.6 ± 0.4 Å for ^14^Hst5. The dissipation values during peptide incubation increase to approximately 0.6 × 10^–6^–0.7 × 10^–6^, indicative of a less rigid system than ^24^Hst5 at this experimental step. However, upon rinsing, this value returns to zero, and therefore, the peptide can be assumed to affect the SLB similarly to ^24^Hst5. Data obtained for ^48^Hst5 indicates a slight time dependence during both incubation and rinsing, where the frequency shift is limited to a few Hz during incubation. However, the increase in frequency during rinsing for the ^48^Hst5 in comparison with both ^24^Hst5 and ^14^Hst5 indicates a different interaction behavior and/or molecular organization might occur. The shift in frequency reached values of Δ*F*_11_/11 = −10.4 and −4.3 Hz, before and after rinsing for ^48^Hst5, respectively, resulting in a peptide layer thickness of *t*^*QCM*^ = 5.7 ± 0.3 Å after rinsing. The dissipation reaches almost 1 × 10^–6^ before rinsing, and upon rinsing, it converges to 0.2 × 10^–6^. Hence, ^48^Hst5 forms a less rigid layer than both ^24^Hst5 and ^14^Hst5. Thus, this indicates a different interaction behavior or molecular organization of this peptide compared to the other two, as already suggested by NR. While the values of *D*_*gap*_ and *t*^*QCM*^ are, for all three samples, of the same order of magnitude, their direct comparison is not trivial because of the intrinsic differences between the NR and QCM-D measuring principles. While the sensitivity of NR in quantifying molecular species in a given region of the sample decreases with increasing hydration, a condition met for the highly hydrated cushion, QCM-D is sensitive only to the net balance of adsorbed and desorbed masses. The determination of *t*^*QCM*^ might be biased by the removal of lipid molecules. However, the very low value of both equivalent thicknesses indicates that the amount of peptide molecules interacting with the SLB is extremely limited. In the case of ^48^Hst5 it is worth noting that the system is slightly less hydrated, as can be seen in Figure 2. At the same time, *t*^*QCM*^ and *D*_*gap*_ are, for this sample, almost identical. These observations could indicate a flatter adsorption of the peptide within the cushion.

#### 4.2.2 The Impact of Salt Concentration

**Figure 3:**
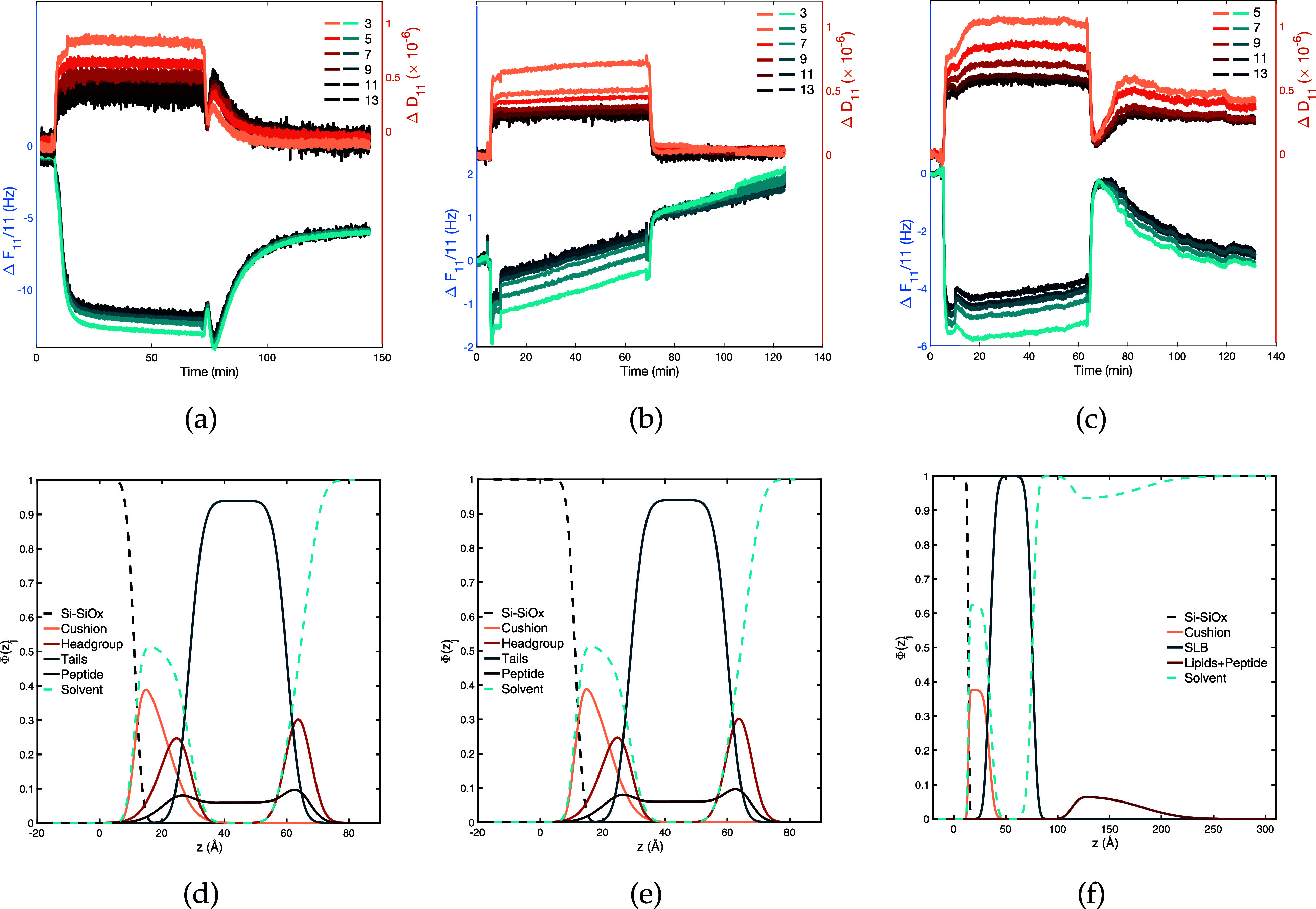
Quartz-crystal microbalance with dissipation monitoring data (frequency shifts, Δ*F*_*n*_/*n*, and dissipation factors, Δ*D*) obtained for (a) ^14^Hst5, (b) ^24^Hst5, and (c) ^48^Hst5 at 150 mM NaCl. For (c), the third overtone was removed since it showed inconsistent trends due to the instability of the instrument. Volume fraction profile components, ϕ_*j*_(*z*), for (d) ^14^Hst5, (e) ^24^Hst5, and (f) ^48^Hst5 obtained from the analysis of neutron reflectometry (NR) data. Data were collected in 150 mM NaCl for ^14^Hst5 and ^48^Hst5, and in 140 mM NaCl for ^24^Hst5.

In a previous study,^8^ we investigated the interaction of ^24^Hst5 with a PC:PS 9:1 SLB, using also partially deuterated POPC and POPS lipids, as _31_PC:d_31_PS 9:1, at 10, 80, and 140 mM NaCl using NR. The results indicated no interaction for the higher salt concentrations. QCM-D measurements, see Figure 3b, show negligible peptide adsorption upon injection; however, during incubation, the frequency increases above zero, which implies that a mass, deviating from the peptide molecular weight, is removed. This is further observed upon rinsing. The increase in frequency is minimal and could originate from minor reorganizations in the system upon interaction of the peptide with the bilayer; however, it is important to stress that this effect is very minor. The dissipation increases upon injection of the peptide and stabilizes, whereas the frequency increases. Hence, the possible removal of lipids does not affect the rigidity of the adsorbed layer, and the dissipation is not decreased until rinsing is initiated. The splitting between the overtones, as shown in Figure 3b, is less than expected upon lipid removal since the latter would introduce a significant amount of water into the system. The data indicated that there might be a negligible, reversible adsorption of the peptide that could rearrange the lipid bilayer with subsequent release. The effect is insignificant, as noted in the low-frequency shift, and impossible to detect in NR. Both the shorter ^14^Hst5 and the longer ^48^Hst5 show a clear interaction with the lipid bilayer at higher NaCl concentration, contrary to ^24^Hst5.

##### 4.2.2.1 Decreased Chain Length

**Figure 4:**
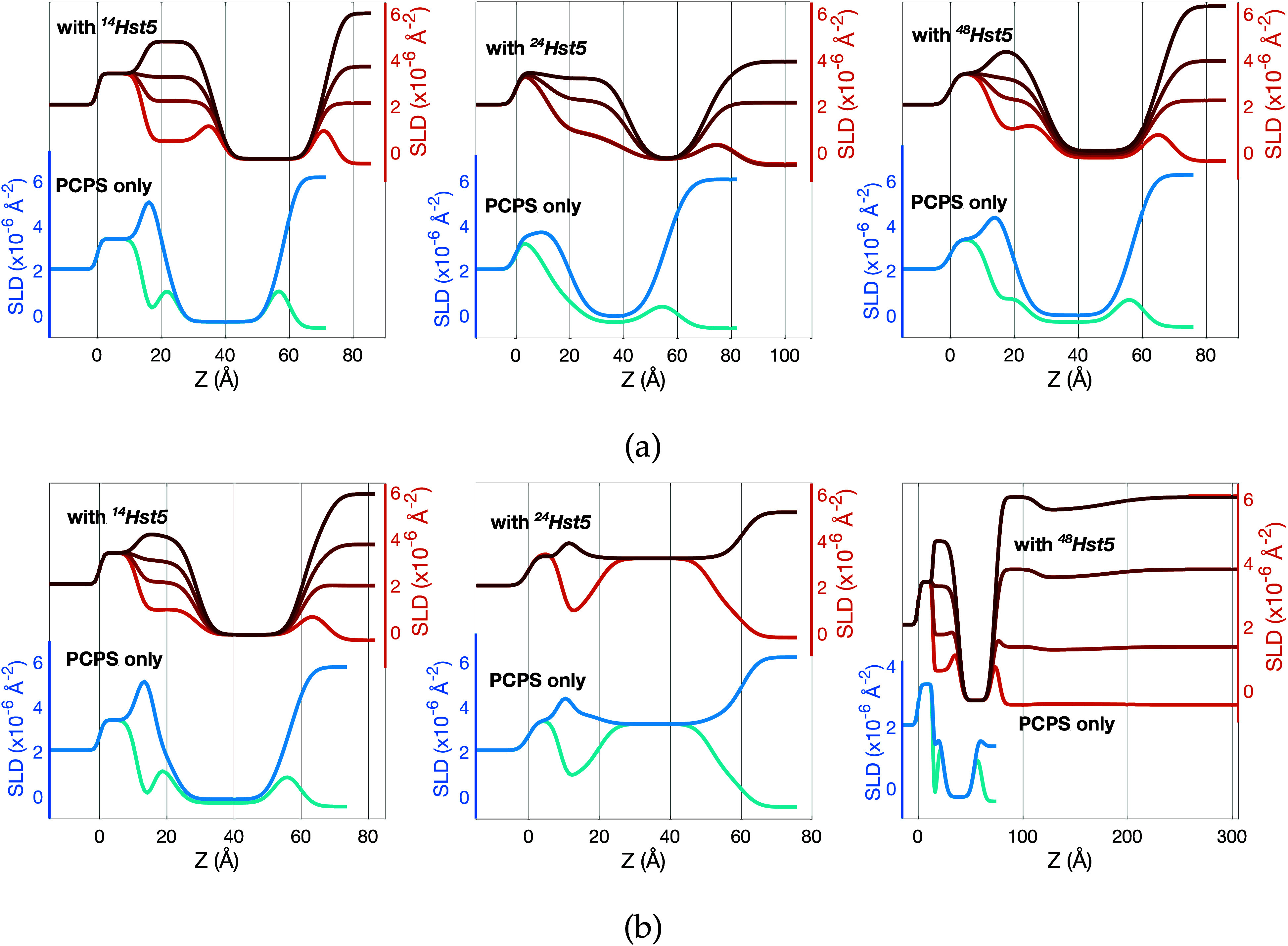
Scattering length density (SLD) profiles of ^14^Hst5 (left), ^24^Hst5 (middle), and ^48^Hst5 (right) at (a) 10 mM NaCl and (b) 150 mM NaCl (140 mM for ^24^Hst5) in comparison to those of the pristine bilayer. SLD profiles are obtained from fitting the data shown in Figures S9–S11. Data for the ^24^Hst5 sample are reproduced from ref (9) for 10 mM NaCl and ref (8) for 140 mM NaCl. The latter was collected using partially deuterated phospholipids and for this reason the center of the SLD profiles reaches ≈3 × 10^–6^ Å^–2^. No differences are expected with respect to the use of protiated lipids.

^14^Hst5 was found, through analysis of NR data, to reside in the gap between the solid substrate and the bilayer, forming a cushion and penetrating both in the head groups and the tail region of the bilayer. By evaluating changes in the SLD values, see Figure 4, the peptide volume fraction resulted in 0.13 ± 0.03 in the headgroup region and 0.06 ± 0.02 in the hydrophobic tails. It should be noted that the interaction of the peptide with the lipid head groups induced a structural reorganization of the lipid molecules, as indicated by the increased volume occupied by water molecules, from 0.1 ± 0.1 to 0.4 ± 0.1 (v/v). Overall, the thickness of the SLB increases, with both headgroup layers increasing from 5.7 ± 0.5 to 7.1 ± 0.7 Å. The increase in thickness is compatible with the inclusion of peptide molecules and the associated water molecules in the absence of a noticeable removal of lipid material, as suggested by the null water volume fraction in the hydrophobic SLB region. As already mentioned, the peptide was also localized between the SLB and the silicon substrate, forming a 10.6 ± 0.6 Å thick cushion, containing 53% of buffer and 47% of peptide, v/v, ± 3%. The lipid content were determined to be 40 ± 10% in the head groups and 94 ± 3% in the tail region, suggesting lipid removal followed by formation of pores in the bilayer. Note that the significant uncertainty in the headgroup composition arises from the limited precision in determining this region’s buffer volume fraction and is summarized in the SLD profiles; see Figure 4b and the VFP presented in Figure 3d. The structural reorganization of the lipid bilayer with increased hydration observed from NR is not visible from QCM-D, which indicates that the sample behaves as a rigid film even after incubation with ^14^Hst5. This is evidenced by the low dissipation and the overlapping normalized frequency shifts shown in Figure 3a. After rinsing, all Δ*F*_*n*_/*n* values stabilized at −5.3 Hz, corresponding to approximately 94 ng cm^–2^ of adsorbed mass in addition to the mass of the SLB before the interaction.

##### 4.2.2.2 Increased Chain Length

The adsorption profile obtained from QCM-D for ^48^Hst5 in 150 mM NaCl, is characterized by the common behavior already described for ^24^Hst5 and ^14^Hst5, with fast adsorption which stabilizes upon incubation; see Figure 3c. Upon rinsing, *t* ≈ 65 min, the frequency and dissipation shifts observed are remarkably different from all the other data reported in the current and previous works for ^24^Hst5 and its variants so far investigated; see Figure 3c.^8,9^ An initial frequency increase and a slight but marked decrease upon continuous rinsing characterize the data. This trend is, to some extent, mirrored in the dissipation curves. However, both frequency and dissipation stabilize more promptly, settling at values on the borderline between those characteristics of a rigid and viscoelastic film regime. Since no additional material is added to the solution while rinsing, this behavior indicates a dynamic process in which some material is first removed from the sensor without being completely detached and removed from the sample solution. Despite applying a constant flow, the removed material can readsorb on the sensor surface. At the end of the measurements, even if maximum stabilization of the frequency was not reached, all Δ*F*_*n*_/*n* were very close to each other and equal to −2.90 ± 0.25 Hz. The analysis of NR data confirmed the different behavior of ^48^Hst5 upon interaction with a PC:PS 9:1 bilayer. As illustrated in Figure 3f, ^48^Hst5 is localized in the cushion region between the solid substrate and the bilayer but also on top of the bilayer, at the interface with the liquid bulk phase. The cushion obtained is characterized by a thickness *t*_*gap*_ = 20 ± 1 Å and a peptide volume fraction of 0.38, leading to an equivalent thickness *D*_*gap*_ = 8 ± 1 Å. The additional layer of peptide formed on the outer SLB surface is very diffuse and highly hydrated, being almost 100 Å thick and composed of 93% buffer, and only 7% ^48^Hst5, v/v%. Given these features and the SLD values of the aqueous media and the peptide, the contribution from this layer is almost invisible in H- and SiM-buffers; see Figure 4b, left-hand panel. This layer, present on top of the bilayer, is most certainly what gives rise to the nonzero dissipation value observed upon rinsing, as opposed to all other investigated systems. Hence, this system is more viscoelastic. Interestingly enough, a correlation between the amount of β-sheets predicted from the CD results and the thickness of the formed cushion, *t*_*gap*_, obtained from NR is observed when ^48^Hst5 is compared to ^24^Hst5. At low salt concentration, ^24^Hst5 displays a larger β-sheet content as well as a larger amount of adsorbed peptide, whereas, at high salt concentration, the β-sheet content is larger in ^48^Hst5, and so also the adsorbed amount.

#### 4.2.3 Computer Simulations

To obtain a molecular understanding, coarse-grained modeling and MC simulations were performed. The focus has been on the peptide residing within the cushion. For this purpose, the cushion is modeled as a slit of two solid surfaces, where one corresponds to the silica surface and the other the inner headgroups of the lipid bilayer.

**Figure 5:**
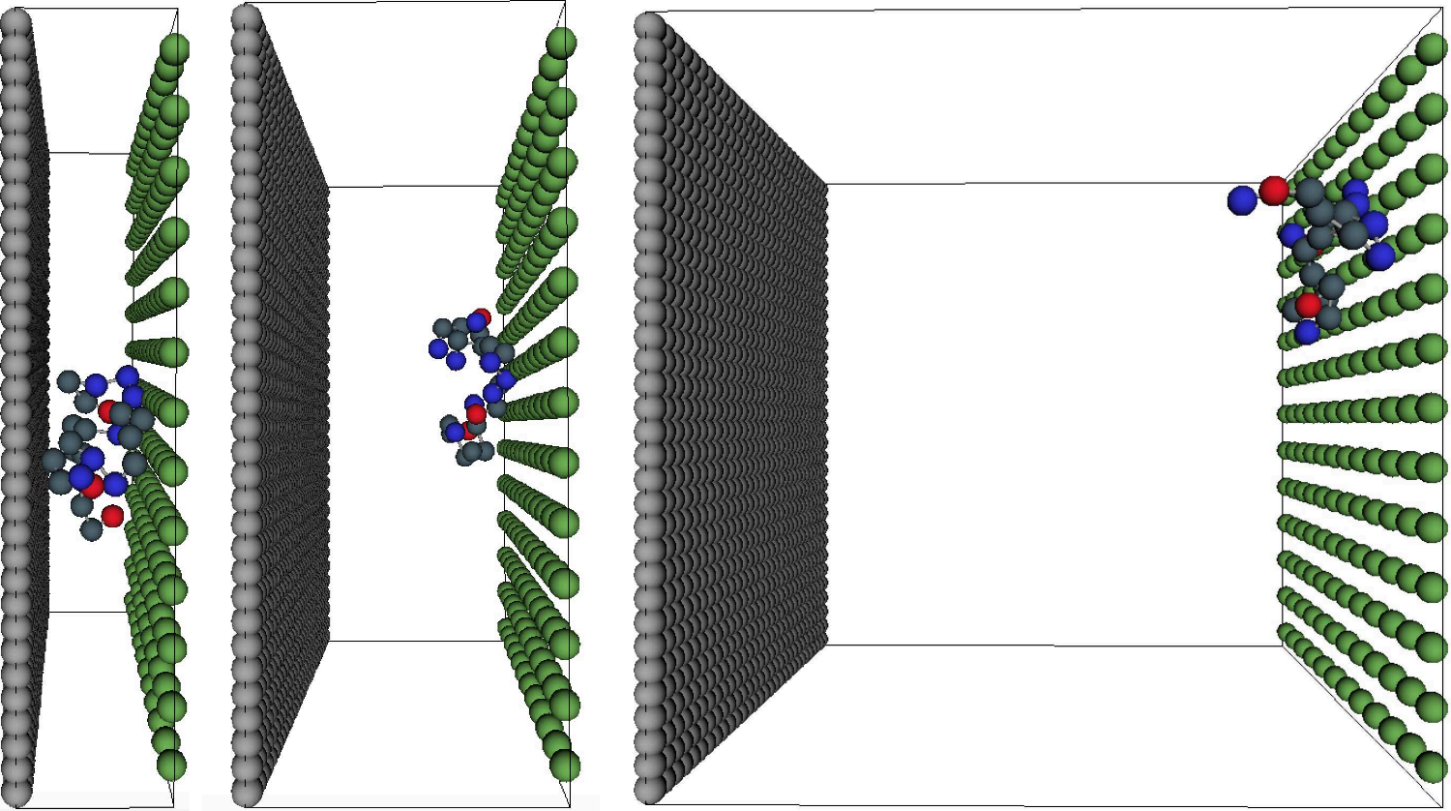
Illustrative snapshots of ^24^Hst5-conformation when residing in the cushion with three different distances, 20, 40, and 100 Å, between the surfaces. The gray surface represents silica, and the green represents the bilayer. The negatively charged amino acids are colored red, the positive ones blue and the uncharged ones are colored blue/gray.

Illustrative snapshots of the model system, and thus ^24^Hst5 in the cushion, for different lengths between the silica surface and the inner headgroups of the SLB are shown in Figure 5. As depicted, ^24^Hst5 prefers to adsorb to the bilayer, and when the cushion becomes narrow, corresponding to the length scales shown in the experiments, ^24^Hst5 is in contact with both surfaces. This is further confirmed by the density distribution of the amino acids in the *z*-direction, displayed in Figure S25. An increase or decrease of the peptide length does not affect this behavior. Hence, we conclude that the ^24^Hst5 system is thermodynamically favored by adsorbing to the bilayer due to the higher surface charge density. Thus, upon adsorption, the system’s free energy decreases due to an increased electrostatic attractive interaction and the accompanying counterion release.

##### 4.2.3.1 Conformational Properties of the Peptides within the Cushion

*R*_*g*_, of ^24^Hst5 remain relatively constant independent of the distance between the surfaces at both salt concentrations investigated; see Table S4, and are similar to those obtained in bulk conditions, as seen in Table S3. Hence, the peptide is slightly compressed by the presence of the surfaces. When the distance between the surfaces is increased, *R*_*g*_ increases. Therefore, expect that the peptide is probably also compressed in the experiments upon interaction with the SLB. Figure S25 shows the number density of all amino acids in the *z*-direction of the box. Notice that the depleted region close to the silica surface is a modeling artifact due to hard-sphere interactions. This is not observed for the bilayer surface as the surface particles are placed within enough distance for the peptide to enter between them. The distribution of amino acids in ^24^Hst5 highly depends on the distance between the surfaces. At a distance of 20 Å between the surfaces, the number density indicates that the peptide is adsorbed to both surfaces, which is further confirmed by the snapshots, shown in Figure 5. This is non-salt-dependent. Increasing the distance between the surfaces to 40 Å changes the number density with higher density toward the bilayer surface. A noticeable salt effect is observed due to electrostatic screening effects. Upon increasing the distance between the surfaces further, to 100 Å, the salt effect remains. In low salt concentrations, the peptide adsorbs only to the bilayer surface. In contrast, at the higher salt concentration, the number density indicates that ^24^Hst5 adsorbs to both surfaces, with a slight preference for the bilayer. From these results, and in comparison with our experiments, we can expect that ^24^Hst5 interacts with both surfaces in the cushion with a reasonably compact conformation.

Interestingly, *R*_*g*_ of ^48^Hst5 decreases when the cushion expands from 20 to 40 Å, and increases again when the surfaces are 100 Å apart, which holds true for both salt concentrations. This could be explained by the fact that the peptide is adsorbed to both surfaces and acts as a bridge, which does not exactly agree with what was observed for ^24^Hst5 and could be a chain-length effect. From the experimental results, we observed a smaller cushion formed for the ^48^Hst5 peptide at 10 mM salt, which we hypothesize is due to a different conformation of the peptides upon adsorption. According to Figure S25 with a 20 Å distance between the surfaces, the ^48^Hst5 peptide does not display a different adsorption conformation in comparison with either ^24^Hst5, or ^14^Hst5 at any of the salt concentrations. The linear charge density of the amino acids in the different peptide chains is almost identical. Hence, this data provides no explanation for the deviating cushion size in the case.

Upon increasing the distance between the surfaces, differences between the peptides arise. With a distance between the surfaces of 40 Å, ^48^Hst5, as opposed to ^24^Hst5, still displays a number density profile indicative of bridging. For this peptide, the difference in number density between the different salt concentrations indicates a higher number density closer to the surfaces in 10 mM salt, whereas, at high salt concentration, the number density is at its highest in the middle of the box. This indicates that fewer amino acids are involved in the adsorption in 150 mM salt. Lastly, ^14^Hst5 actually displays a larger *R*_*g*_ value with surfaces present at 40 and 100 Å in 10 mM NaCl, and 100 Å in 150 mM NaCl, compared to bulk conditions. This could be due to the peptide’s preference to interact with both surfaces and, therefore, be extended to reach both with increasing surface distance and with a 40 Å distance between the surfaces ^14^Hst5 displays a number density profile similar to the one observed for ^24^Hst5 in both salt concentrations. In 10 mM salt, the number density indicates a preference of the peptide to adsorb to the bilayer surfaces, whereas, in 150 mM salt, the density is more smeared with a higher density in the middle region, compared to 10 mM, as shown in Figure S25, middle panel. Upon increasing the distance between the surfaces to 100 Å, the ^14^Hst5 peptide still follows the behavior observed for ^24^Hst5; however, in 10 mM salt for this peptide the number density display some interaction with the silica surface as well. The errors obtained for the values measured for this peptide are quite large, but despite this, the number density close to the silica surface is significantly different from zero. At higher salt concentrations, a very similar curve to the one observed for ^24^Hst5 is observed, indicative of a very similar behavior of the two peptides.

##### 4.2.3.2 The Adsorption Profiles

**Figure 6:**
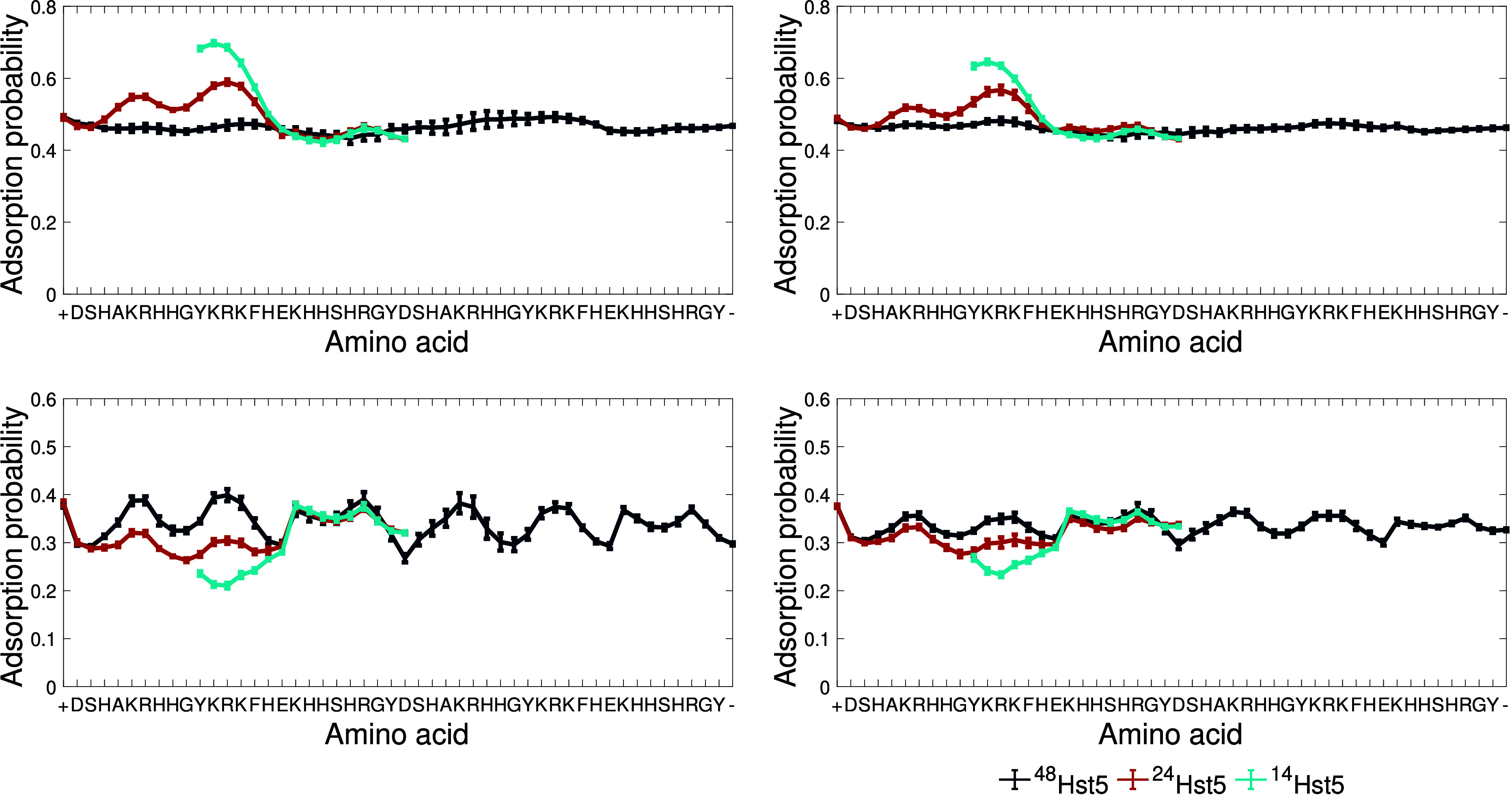
Adsorption profile of the three different peptides to a surface mimicking a bilayer (top) with a total charge of −78*e*, −0.5*e*/point, and a surface mimicking a silica surface (bottom) with a total charge of −49.5*e*/point and −0.05*e*/point at 10 and 150 mM NaCl, respectively. The distance between the surfaces, mimicking a cushion, is 20 Å.

As a complement to the conformational properties of the three peptides, the adsorption profiles to the two surfaces were investigated, as shown in Figure 6, for 20 Å to mimic the environment within the cushion best. It is observed that the adsorption probabilities are higher for the bilayer surface, which is explained by its higher overall net charge and higher charge particle. ^24^Hst5 displays two peaks, mainly residues K and R. In the case of the silica surface, the positively charged N-terminus is the most probable residue to adsorb. The adsorption probability is slightly decreased for both surfaces when the salt concentration is increased from 10 to 150 mM NaCl; however, the shape of the adsorption profiles are the same. These results, together with the results previously discussed, indicate that the peptide bridges the two surfaces. For ^48^Hst5, the adsorption profile to the bilayer is relatively flat, and is lower in both salt concentrations in comparison to ^24^Hst5. The adsorption profile to the silica surface, on the contrary, displays several minima and maxima, where, as for ^24^Hst5, the maxima are centered around the amino acids K and R. Here, the adsorption probability is higher compared to ^24^Hst5 in both salt concentrations. Again, as previously discussed, these results indicate that the peptide bridges the two surfaces. Lastly, ^14^Hst5 shows the highest adsorption probability of the three peptides to the bilayer surface in both salt concentrations. As for the other two peptides, the maximal adsorption probability is found near amino acids K and R, whereas the lowest adsorption probability is shown on the silica surface. For this short peptide, the adsorption probability is mirrored between the surfaces, and since the peptide is very short, it is probably stretched between these surfaces to be able to adsorb to both of them. The reader should note that since explicit charges are applied on the surfaces, this distance, about the distribution of charged amino acids in the primary sequence, will play a role. Hence, the result is system specific, as *in vivo*.

### 4.3 Bioinformatic Predictors and Charged Patches

In a recent paper it was shown that ^24^Hst5 possesses the well-known HExxH zinc motif and a second motif, HAKRHH, which is important in forming zinc-induced oligomers.^49^ In addition to this, it was shown in a previous study by Kurut et al. that exchanging amino acids 12 to 14, thus KRK, with uncharged glycine (G) completely eliminated the adsorption of the peptide.^50^

We hypothesize that these motifs and charged patches could act as NLS. To investigate this further, three online predictors were used, , NLStradamus,^51^ PSORT II,^52^ DeepLoc-2.0^53^, and are presented in Table 1. In addition to this investigation, predictors for CPP and antifungal properties were also used, to get a better understanding of how the chain length is affecting the biological properties of the peptide. Therefore, an additional four predictors were utilized for CPP prediction,^54−57^ and three to predict the antifungal effect of the peptides.^58−60^ According to the NLStradamus, ^24^Hst5 contains one NLS, where the length of the NLS depends on the cutoff used in the prediction. However, according to PSORT II, none of the three categories of NLSs are included in this peptide. DeepLoc-2.0 gave a 93% probability that ^24^Hst5 is located in the nucleus. Figure S29 displays which amino acids in the sequence were most important in the prediction obtained from DeepLoc-2.0, showing that the C-terminus contributes the most. Even though these three predictors do not agree, we can conclude that amino acid patches are similar to NLSs and may play an important part in the ability of ^24^Hst5 to translocate the bilayer. The patches consist largely of the amino acids K and R, positively charged at physiological pH. They could help facilitate the initial interaction with the bilayer, followed by the translocation. This could explain why the peptide loses the ability to translocate the bilayer at higher salt concentrations since the electrostatic interactions between these amino acids and the bilayer/silica surface are screened. Out of the four predictors used to predict if ^24^Hst5 is a CPP, three of them did. Using different lengths of the sequence, 3–9 sequences for CPP were found using CellPPD,^57^ and according to MLCPP 2.0,^55^^24^Hst5 has a high uptake efficiency. Regarding its antifungal effect, ^24^Hst5 is known to be active against primarily *C. albicans*,^2,18,19,61−64^ the predictors are in line with those results. They all predict ^24^Hst5 to be antifungal. The predictor Antifungipept^60^ gives an Antifungal Index (AFI), which gives information about the overall antifungal capability of the peptide, where a lower value indicates a stronger broad-spectrum antifungal activity and a higher value suggests weaker efficacy. For ^24^Hst5, this value is 7.25 μM, and the peptide is predicted to be most active against *C. albicans*, in line with previous results.

**Table 1:** Predictions if the Three Peptides Contain Nuclear Localization Sequences^a^

Predictor	^14^Hst5	^24^Hst5	^48^Hst5
NLS			
NLStradamus (0.5/0.55 cutoff)	No NLS	res. 7–17/res. 11–13	res. 5–45
PSORT II			
DeepLoc-2.0	Nucleus 78%	Nucleus 93%	Nucleus 68%
			Extracellular 72%
CPP			
BChemRF-CPPred	Non-CPP 59%	Non-CPP 68%	CPP 80%
MLCPP-2.0	CPP 85%	CPP 75%	CPP 55%
	Low uptake efficiency (48%)	High uptake efficiency (66%)	High uptake efficiency (80%)
C2Pred	CPP 0.97	CPP 0.91	CPP 0.92
CellPPD 10 aa fragment	One CPP-sequence	Nine CPP-sequences	18 CPP-sequences
CellPPD 15 aa fragment		Six CPP-sequences	14 CPP-sequences
CellPPD 20 aa fragment		Three CPP-sequences	Ten CPP-sequences
Antifungal Effect			
AntiFP	Antifungal (score: 0.17)	Antifungal (score: 1.0)	Antifungal (score: 1.0)
AFPtransferPred	Antifungal (score: 0.98)	Antifungal (score: 0.95)	Antifungal (score: 0.82)
Antifungipept	Antifungal (prob: 100%)	Antifungal (prob 100%)	Antifungal (prob. 98.8%)
	AFI 16.57 μM	AFI 7.25 μM	AFI 5.95 μM

a Made by the three online predictors NLStradamus,^51^ PSORT II,^52^ and DeepLoc-2.0.^53^ Predictions regarding cell-penetrating peptides were performed by the online tools BChemRF-CPPred,^54^ using version 2.0, and the FC-3 Feature Composition, MLCPP-2.0,^55^ C2Pred,^56^ and CellPPD,^57^ which were used with the SVM prediction method, and a threshold of 0.0. The antifungal effect was predicted by online tools AntiFP,^58^ AFPtranferPred,^59^ and Antifungipept.^60^

For ^14^Hst5 there are no indications of NLS according to NLStradamus or PSORT II, while DeepLoc-2.0 predicts the peptide to reside within the nucleus with a probability of 78%. ^14^Hst5 is predicted to lack signals to localize the nucleus by two predictors entirely and has a lower probability of residing within the nucleus than ^24^Hst5. In the experiments, we have seen that ^14^Hst5 is equally good at translocating the bilayer at low salt concentration and even better than ^24^Hst5 at high salt concentration, which indicates that instead charged patches similar to NLSs are involved. These results are also in line with the CPP predictors, where the same three predictors predict ^14^Hst5 to be a CPP as ^24^Hst5, where ^14^Hst5 got a higher probability for all three. It was predicted to have a low uptake efficiency,compared to high for ^24^Hst5, by MLCPP-2.0.^55^ Hence, ^14^Hst5 seem to overall be better at translocating the cell membrane than ^24^Hst5, as shown by our experimental results. Regarding the predicted antifungal effect, ^14^Hst5 is by all predictors given scores indicating lower efficacy compared to ^24^Hst5, and the AFI value does not indicate it to be particularly effective against any of the considered species. A large proportion of ^48^Hst5 is predicted to be an NLS, both indicated by NLStradamus and PSORT II, as shown in Table 1. However, according to the DeepLoc-2.0, the peptide is only predicted to be in the nucleus with a 68% probability, and the results showed an even higher probability that the peptide is extracellular, 72%. This peptide is, in contrast with the other two, predicted to be a CPP by all three predictors and to have a high uptake efficiency, which is reasonable, as both this peptide, and ^14^Hst5, are translocated over the lipid bilayer at high salt concentration, where ^24^Hst5 does not interact at all. In addition to this, the antifungal effect seems to be preserved for doubling the length of the original ^24^Hst5 sequence, according to the used predictors, which all only show a slightly lower score compared to ^24^Hst5. The AFI value predicted^60^ is lower than ^24^Hst5, meaning this peptide has higher efficacy against more species. To conclude, even if the three peptides originate from the same primary sequence, the different lengths give rise to a different pattern regarding NLSs. In contrast, the predictions regarding CPP and antifungal effect are quite similar for all three.

#### 4.3.1 Evaluation by Computer Simulations

The impact of the charged patches for ^14^Hst5, ^24^Hst5, and ^48^Hst5 has been further studied by removing the positive charge of a few selected residues, focusing on the charged patches, see Table 2. For this purpose, a system containing a surface mimicking the headgroups of the bilayer at low ionic strength was used.

**Table 2:** Alterations Performed on the Different Peptides^a^

Peptide	Altered amino acid
^14^Hst5	R2
^24^Hst5	R6
	R12
	R6R12
^48^Hst5	R12
	R12R22
	R12R36
	R22R36

a The positive charge was removed on the indicated amino acids.

For ^24^Hst5, the most considerable effect on the adsorption behavior is observed when the salt concentration is altered, rather than the alterations on the amino acid sequence, where the adsorption probability is significantly decreased upon increased salt concentration, as shown in Figure S28 (middle). The overall adsorption profile is maintained, even though the adsorption probability is highly decreased, where the positively charged amino acids show a higher adsorption probability. These amino acids are fairly evenly distributed over the sequence, as shown in Figure 1, however, the highest adsorption probability is observed for the first 15 residues, and the C-terminus show a significantly lower adsorption probability. For the amino acid alteration, there are two patches more likely to adsorb to the surface, namely, around residue 6 and residue 12, as shown in Figure S28 (middle). Removing the charge on either or both of these residues diminishes the adsorption probability for the nearest neighbors, but the overall shape of the probability curve is maintained. Snapshots for ^24^Hst5 are shown in Figure S26.

Results obtained from the simulations of ^48^Hst5 are presented in Figure S28 (right). The most significant effect on the adsorption probability is, as for ^24^Hst5, obtained when the salt concentration is increased from 10 to 150 mM, and the effect observed for the different amino acids alterations is much smaller. However, an apparent decrease in the adsorption probability of the amino acids in the vicinity of the altered one is observed from the alterations, similar to what was observed for ^24^Hst5. The alterations made to the chain do not display a different adsorption shape from this data. Representative snapshots from the simulations, Figure S27, display slightly more loop formation upon adsorption in the altered peptides, whereas the original chain displays slightly more compact adsorption. The snapshots further confirm the lower adsorption probability in 150 mM NaCl, from which it is clear that the adsorption is weaker, and the peptide even desorbs from the surface.

For ^14^Hst5, only one alteration to the amino acid sequence was performed due to its limited length, where the positive charge of the arginine in position two was removed. As with both ^24^Hst5 and ^48^Hst5, the most significant effect on the adsorption profile was observed when changing the salt concentration in the system, as shown in Figure S28 (left). The adsorption probability of this peptide is, however, less affected by increasing the salt concentration from 10 to 150 mM NaCl compared to the other two peptides, in agreement with the experimental results.

## 5 Conclusions

**Figure 7:**
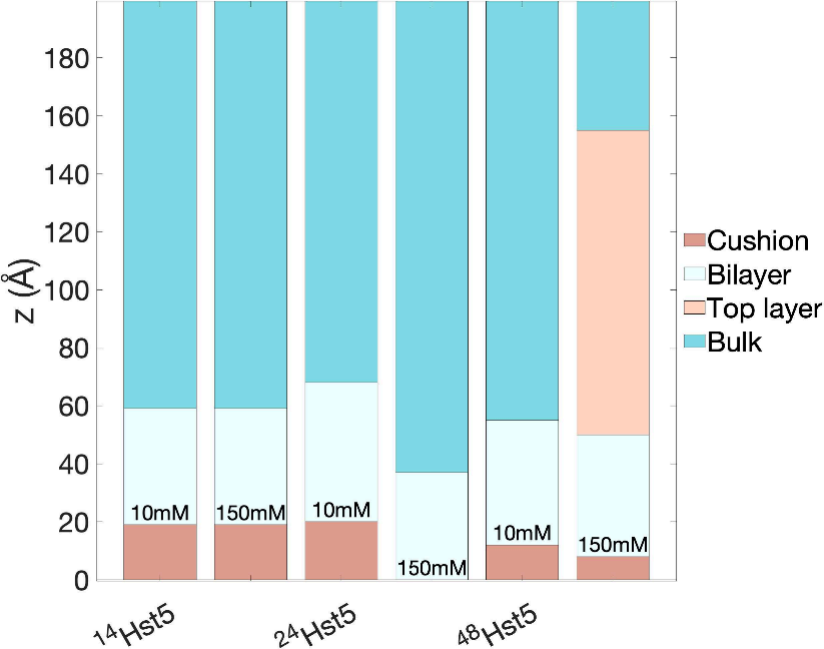
Illustrative representation of the peptide-bilayer systems displaying the thickness of each layer, as determined from fitting NR data.

The conclusion of this study is summarized in Figure 7, which shows an illustrative representation of the investigated peptide-bilayer systems. At low ionic strength, the shorter and longer peptides translocate through the bilayer and form a cushion in line with the behavior of ^24^Hst5. At higher ionic strength, resembling physiological conditions, there is a discrepancy in the results, where the shorter peptide, ^14^Hst5, is capable to translocate across the bilayer and form a cushion, contrary to ^24^Hst5, where no peptide is found within or in the vicinity of the bilayer. The longer peptide chain, ^48^Hst5, seems to interact with the lipids and, in addition to forming a cushion, also accumulates on the top of the bilayer. From these observations, we hypothesize that short- and long-ranged electrostatic interactions play a crucial role in the interaction between the peptide and the bilayer, and they depend on the linear charge density through the primary sequence and the charged patches. We also notice that an increased electrostatic screening plays a role for ^24^Hst5 but not for the other peptides. Moreover, the reason that the peptides are able to form a cushion is the counterion release and the increased osmotic pressure after peptide translocation through the bilayer and its adsorption to the inner lipid headgroups, in combination with excluded volume effects. By being able to control, predict, and tune the peptide translocation ability and the properties of the resulting cushion through the electrostatic interactions, we open up new application areas, for example, in pharmacology and drug development. Finally, we hypothesize that ^24^Hst5 and the shorter variant can also be used as a cargo molecule for the active ingredients in drugs, which is an ongoing study.

## Supporting Information Available

Additional CD fits, data obtained from SAXS, reflectivity curves obtained from NR, additional overtones of QCM-D data, additional simulation data, including snapshots, and further information from the NLS predictors (PDF)
