# Supplementary material for: Translocation of Antimicrobial Peptides across Model Membranes: The Role of Peptide Chain Length
Source: Mol Pharm. 2024 Jul 12;21(8):4082–97. doi: 10.1021/acs.molpharmaceut.4c00450 (PMC11304388; doi:10.1021/acs.molpharmaceut.4c00450)
Supplement: Supplementary file 1 — mp4c00450_si_002.pdf [file mp4c00450_si_002.pdf]

Supporting information: Translocation of Antimicrobial Peptides Across Model Membranes: The Role of Peptide Chain Length

Amanda E. Skog,[†] Nicolò Paracini,[‡] Yuri Gerelli,[¶,§] and Marie Skepö[*,†,‖]

†*Division of Computational Chemistry, Department of Chemistry, Lund University, P.O. Box 124, SE-221 00, Lund, Sweden*

‡*Institut Laue-Langevin, 71 Avenue des Martyrs, 38000 Grenoble, France*

¶*Institute for Complex Systems - National Research Council (ISC-CNR), Piazzale Aldo Moro 2, 00185 Roma, Italy*

§*Department of Physics, Sapienza University of Rome, Piazzale Aldo Moro 2, 00185 Roma, Italy*

‖*NanoLund, Lund University, Box 118, 22100 Lund, Sweden*

E-mail: marie.skepo@compchem.lu.se

S1 Circular Dichroism Data and Fitting

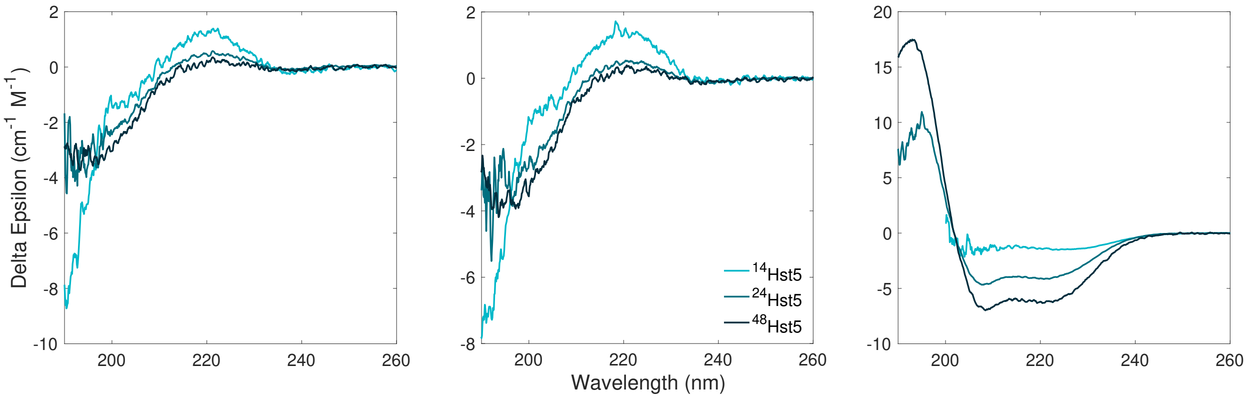

Figure S1: Circular dichroism (CD) data obtained for ^{14}Hst5, ^{24}Hst5, and ^{48}Hst5 in 10 mM (left), 150 mM (middle), and TFE (right), respectively.

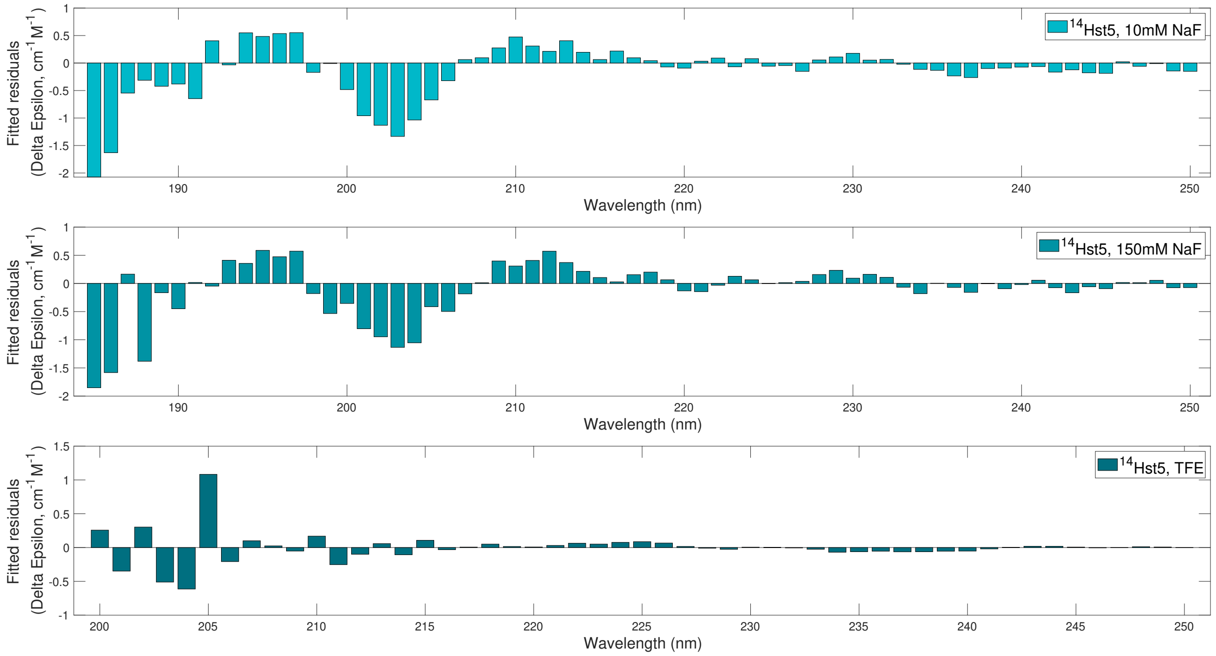

Figure S2: Fitted residuals using BeStSel for circular dichroism (CD) spectra of ^{14}Hst5 in 10 mMNaCl, 20 mMTRIS at pH 7.4 (top), 150 mMNaCl, 20 mMTRIS at pH 7.4 (middle), and TFE (bottom). The CD curves are graphically displayed in Figure S1.

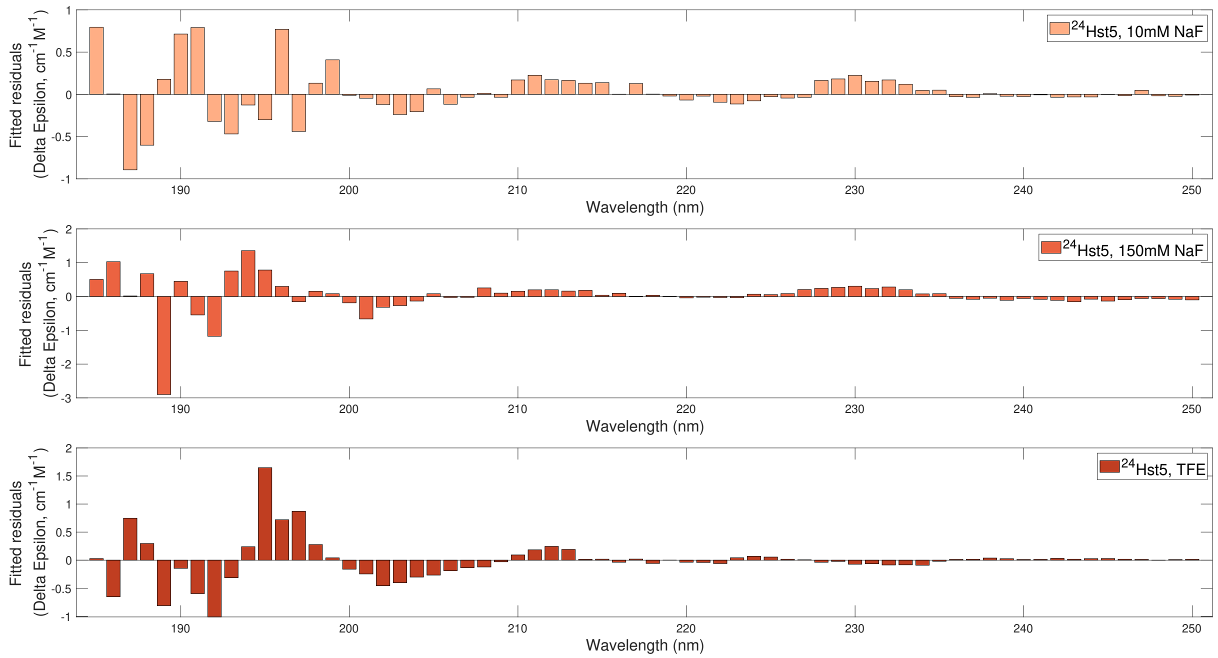

Figure S3: Fitted residuals using BeStSel for circular dichroism (CD) spectra of ^{24}Hst5 in 10 mMNaCl, 20 mMTRIS at pH 7.4 (top), 150 mMNaCl, 20 mMTRIS at pH 7.4 (middle), and TFE (bottom). The CD curves are graphically displayed in Figure S1.

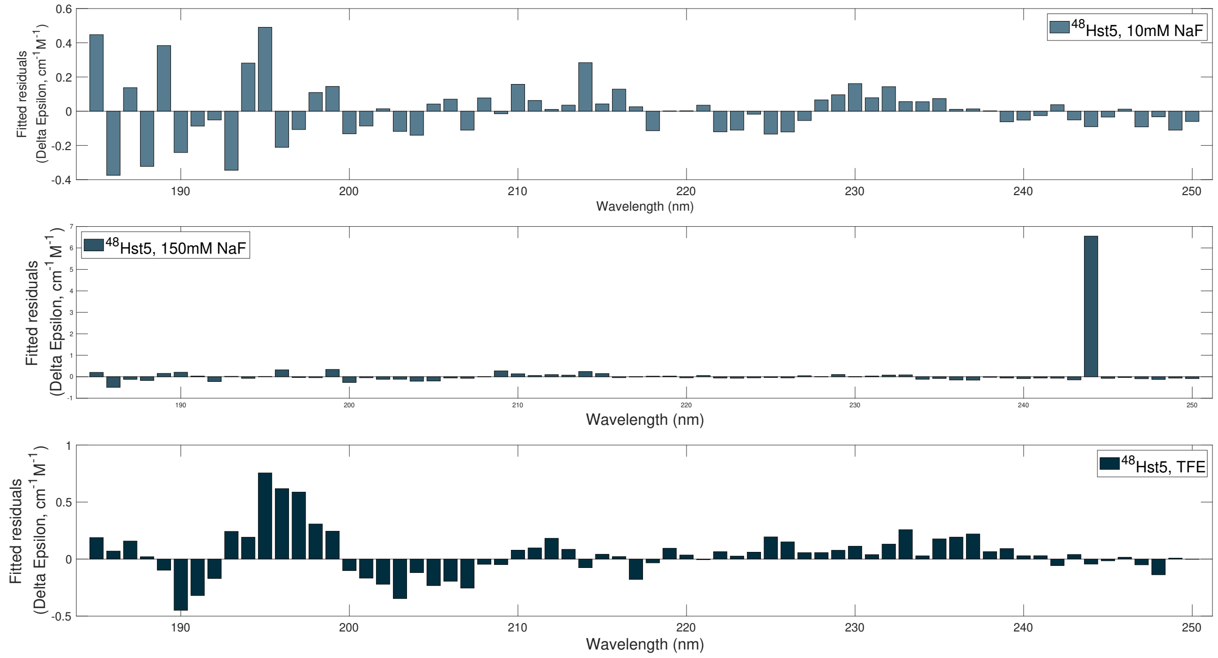

Figure S4: Fitted residuals using BeStSel for circular dichroism (CD) spectra of ^{48}Hst5 in 10 mMNaCl, 20 mMTRIS at pH 7.4 (top), 150 mMNaCl, 20 mMTRIS at pH 7.4 (middle), and TFE (bottom). The CD curves are graphically displayed in Figure S1.

Table S1: Fitting of the circular dichroism data to get the amount of different secondary structures of the peptides in aqueous buffer or 2,2,2-trifluoroethanol (TFE). Prediction performed using SELCON3, *SELCON2 (when no SLECON3 could be found) and BeStSel

Peptide	α-helix [%]			β-sheet [%]			Unordered [%]		
	10 mM	150 mM	TFE	10 mM	150 mM	TFE	10 mM	150 mM	TFE
^{14}**Hst5**									
SELCON3, *SELCON2	*11	*10	*29	*25	*23	*21	*65	*66	*51
BeStSel	12	11	7	17	17	18	71	72	76
^{24}**Hst5**									
SELCON3, *SELCON2	*11	5	49	*31	34	13	*56	47	37
BeStSel	2	14	46	32	23	10	66	64	44
^{48}**Hst5**									
SELCON3, *SELCON2	*11	*11	61	*35	*34	11	*54	*55	26
BeStSel	5	5	62	28	29	5	67	66	23

S2 Small Angle X-ray Scattering Data

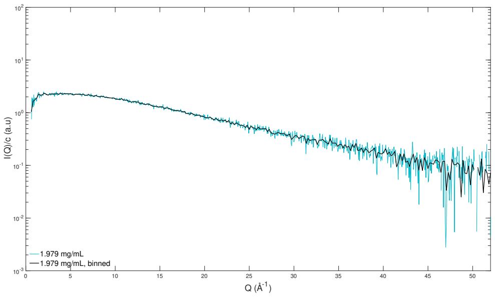

(a)

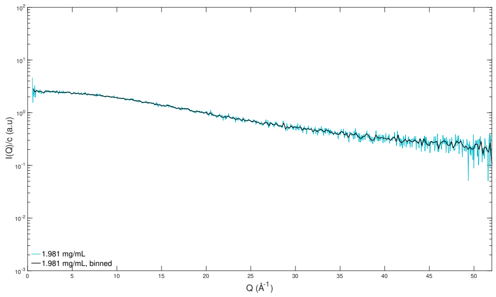

(b)

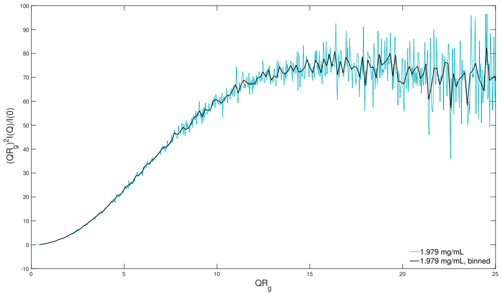

(c)

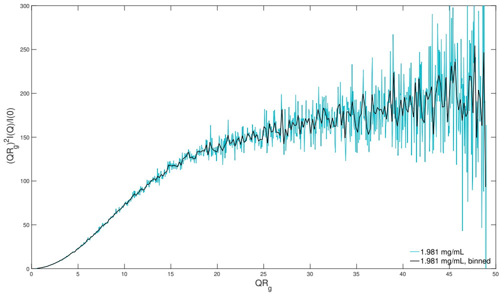

(d)

Figure S5: Comparison of binned small angle X-ray scattering (SAXS) data with non-binned SAXS for ^{14}Hst5. In the binned data, four points were averaged into one. The data obtained in 10 mM NaCl is shown in (a) for the intensity curve and (c) for the normalized Kratky plot. The data obtained in 150 mM NaCl is shown in (b) for the intensity curve and (d) for the normalized Kratky plot.

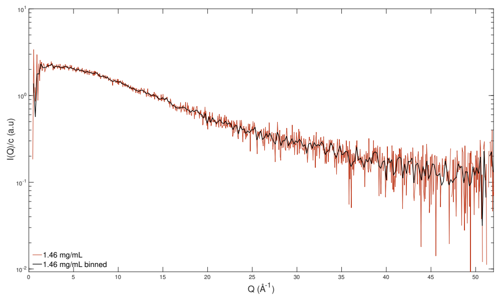

(a)

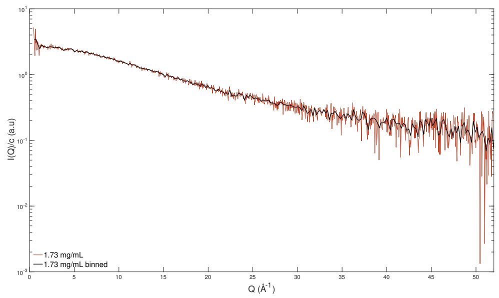

(b)

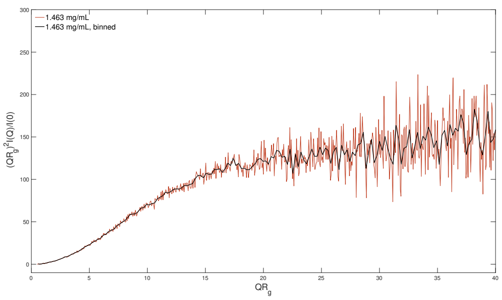

(c)

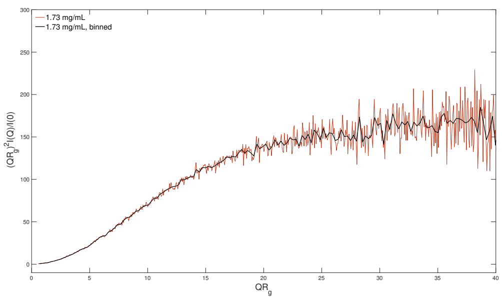

(d)

Figure S6: Comparison of binned small angle X-ray scattering (SAXS) data with non-binned SAXS data for ^{24}Hst5. In the binned data, four points were averaged into one. The data obtained in 10 mM NaCl is shown in (a) for the intensity curve and (c) for the normalized Kratky plot. The data obtained in 150 mM NaCl is shown in (b) for the intensity curve and (d) for the normalized Kratky plot.

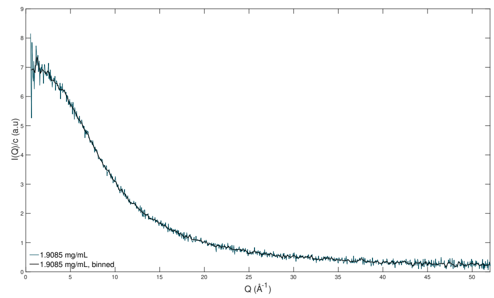

(a)

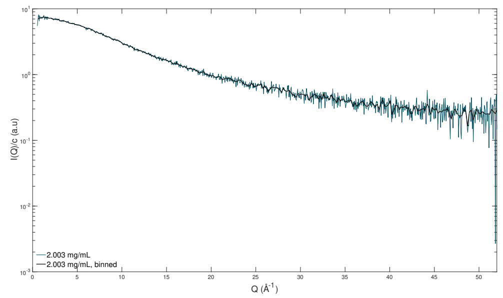

(b)

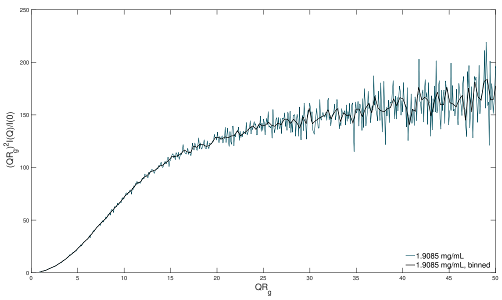

(c)

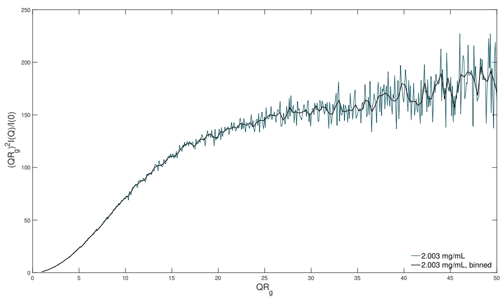

(d)

Figure S7: Comparison of binned small angle X-ray scattering (SAXS) data with non-binned SAXS for ^{48}Hst5. In the binned data, four points were averaged to one. The data obtained in 10 mM NaCl is shown in (a) for the intensity curve and (c) for the normalized Kratky plot. The data obtained in 150 mM NaCl is shown in (b) for the intensity curve and (d) for the normalized Kratky plot.

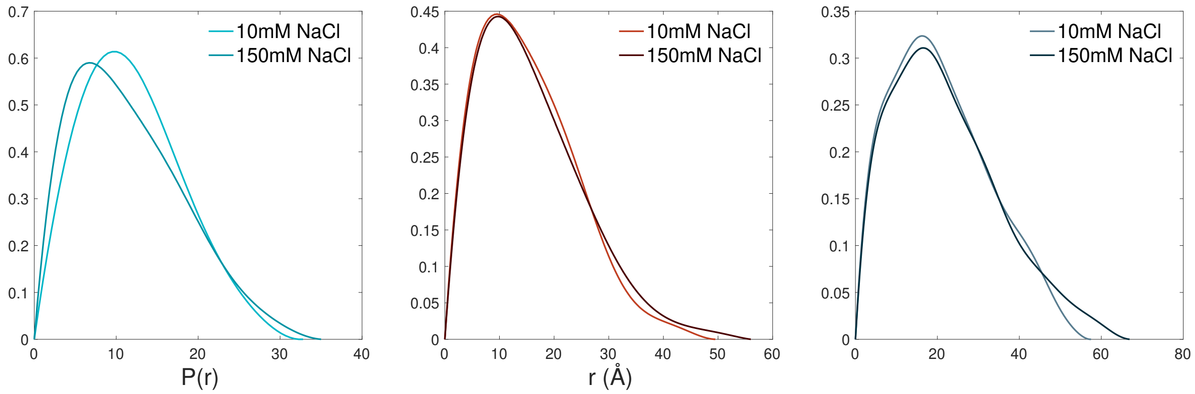

Figure S8: Distance distribution, P(r), plots at 10, and 150 mM NaCl concentration for ^{14}Hst5 (left), ^{24}Hst5 (middle), and ^{48}Hst5 (right).

Table S2: Radius of gyration (R_g), Peak position of the distance distribution curves, P(r), as well as maximum length of the peptide, D_{max} values, determined for all three peptides at 10 mM and 150 mM NaCl. Obtained from fitting small angle X-ray scattering data.

Peptide	Concentration [mg mL^{-1}]		R_g [Å]		P(r) peak position [Å]		D_{max} [Å]	
	10 mM	150 mM	10 mM	150 mM	10 mM	150 mM	10 mM	150 mM
^{14}Hst5	1.979	1.981	7.2 ± 0.5	9.4 ± 0.3	9.8	6.7	32.8	35.0
^{24}Hst5	1.463	1.730	11.3 ± 0.3	12.6 ± 0.3	9.5	9.7	49.5	56.0
^{48}Hst5	1.909	2.003	16.3 ± 0.1	17.2 ± 0.1	16.4	16.5	57.5	66.9

S3 Neutron Reflectometry Data

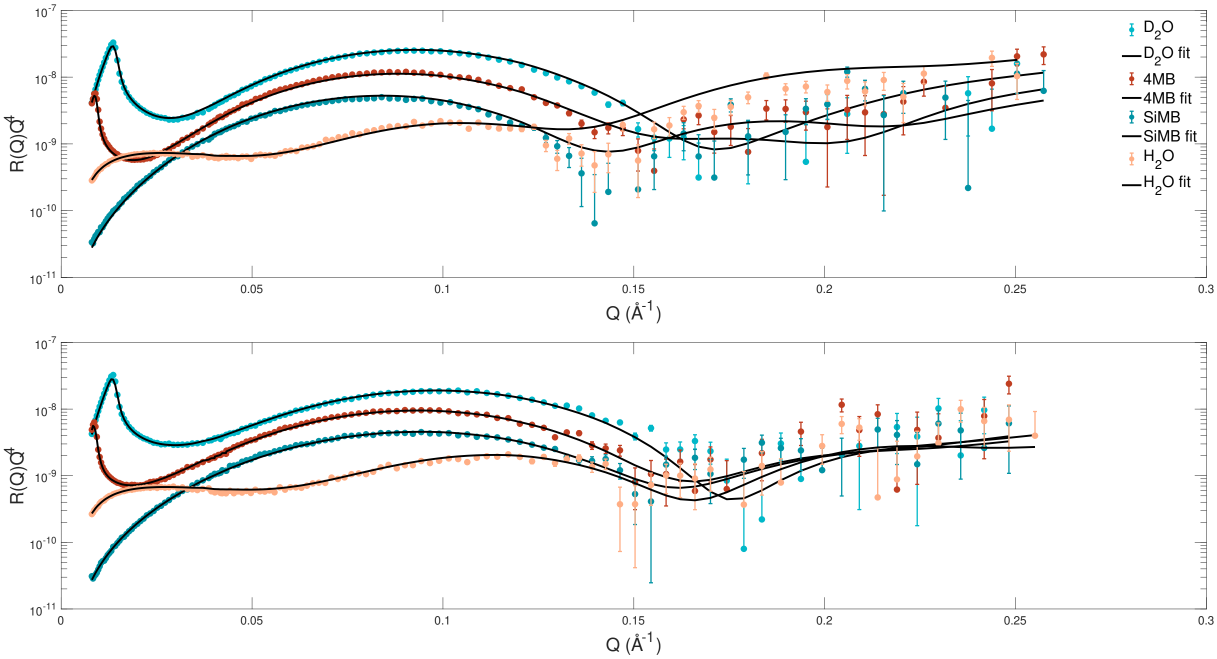

Figure S9: Reflectivity curves for ^{14}Hst5 in 10 mM NaCl buffer (top), and in 150 mM NaCl buffer (bottom) obtained in four contrasts. Included in black are the fitted curves.

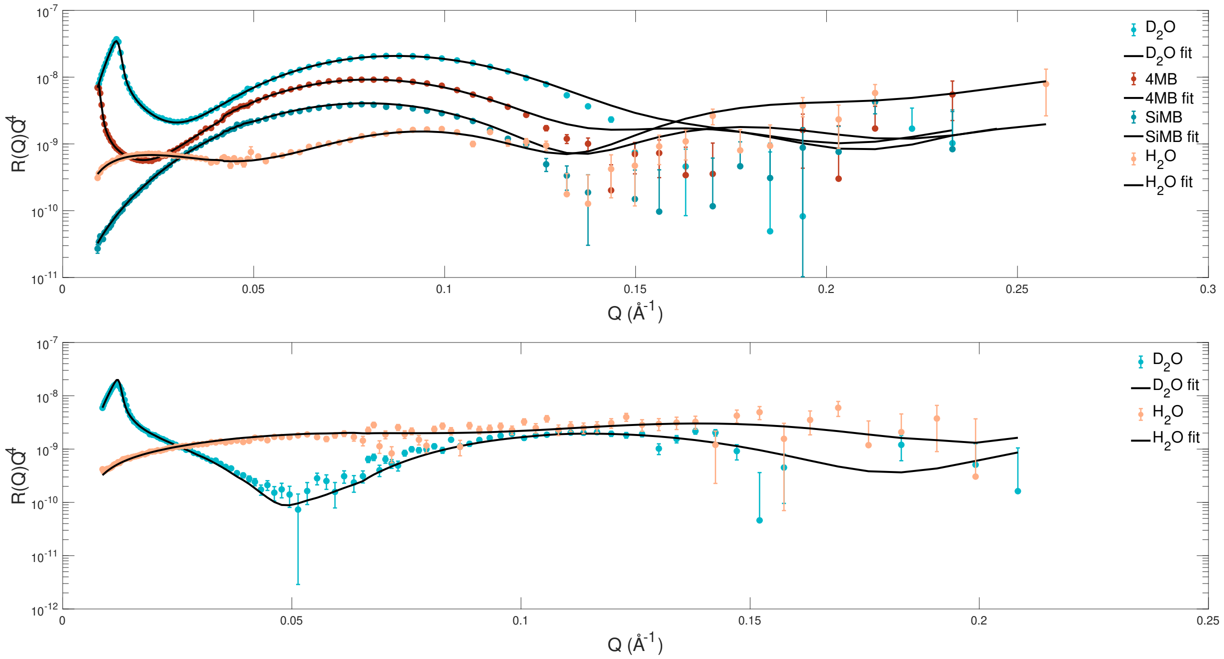

Figure S10: Reflectivity curves for [24]Hst5 in 10 mM NaCl buffer (top), and in 140 mM NaCl buffer (bottom) obtained in four, or two contrasts, respectively. Included in black are the fitted curves.

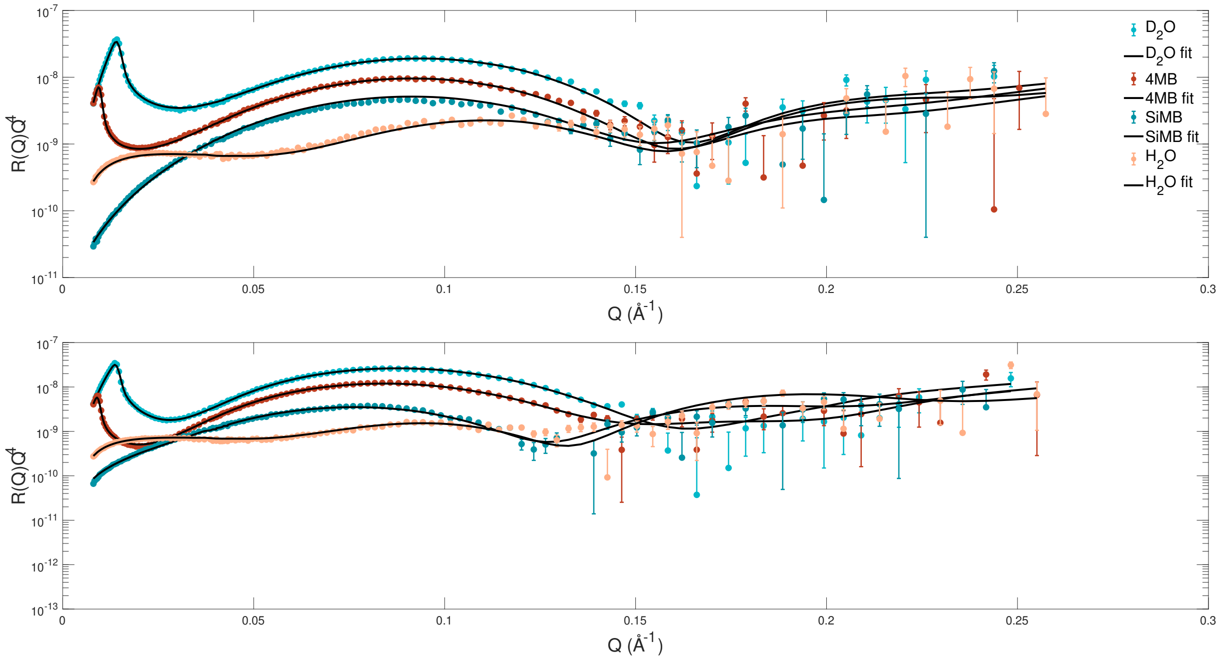

Figure S11: Reflectivity curves for [48]Hst5 in 10 mM NaCl buffer (top), and in 140 mM NaCl buffer (bottom) obtained in four contrasts. Included in black are the fitted curves.

S4 Quartz-Crystal Microbalance with Dissipation monitoring Data

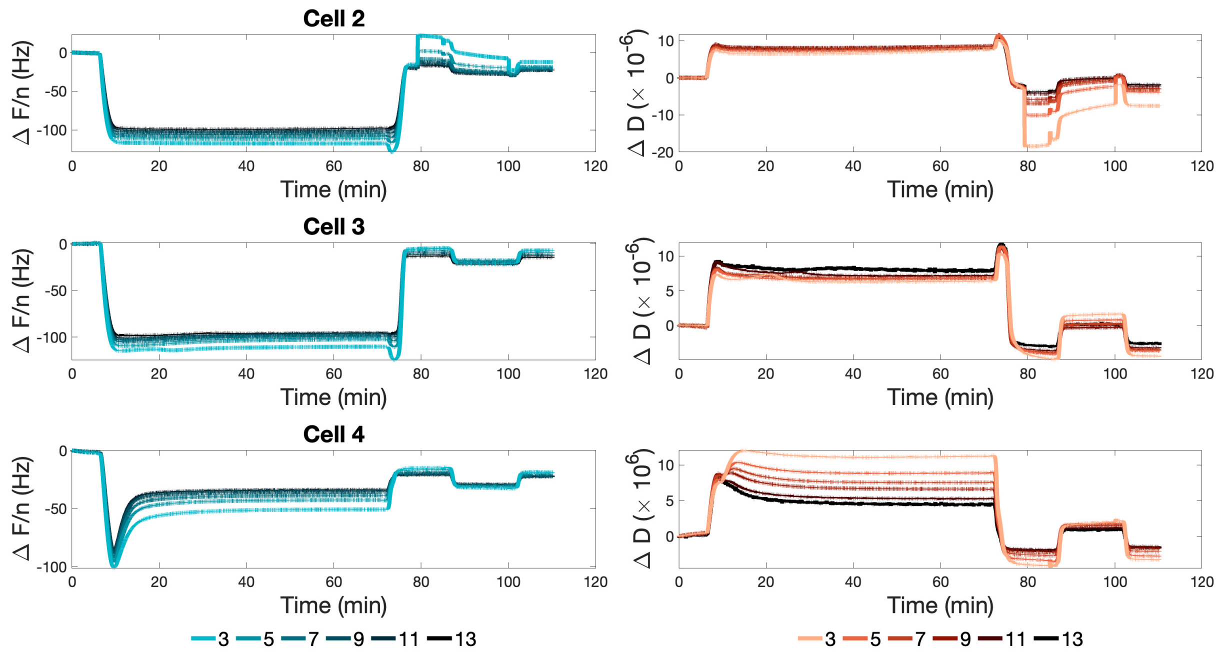

Figure S12: Quartz-crystal microbalance with dissipation monitoring data of the supported lipid bilayer formation to which ^{14}Hst5 in 10 mM NaCl buffer were injected to. All overtones for each measurement cell are presented.

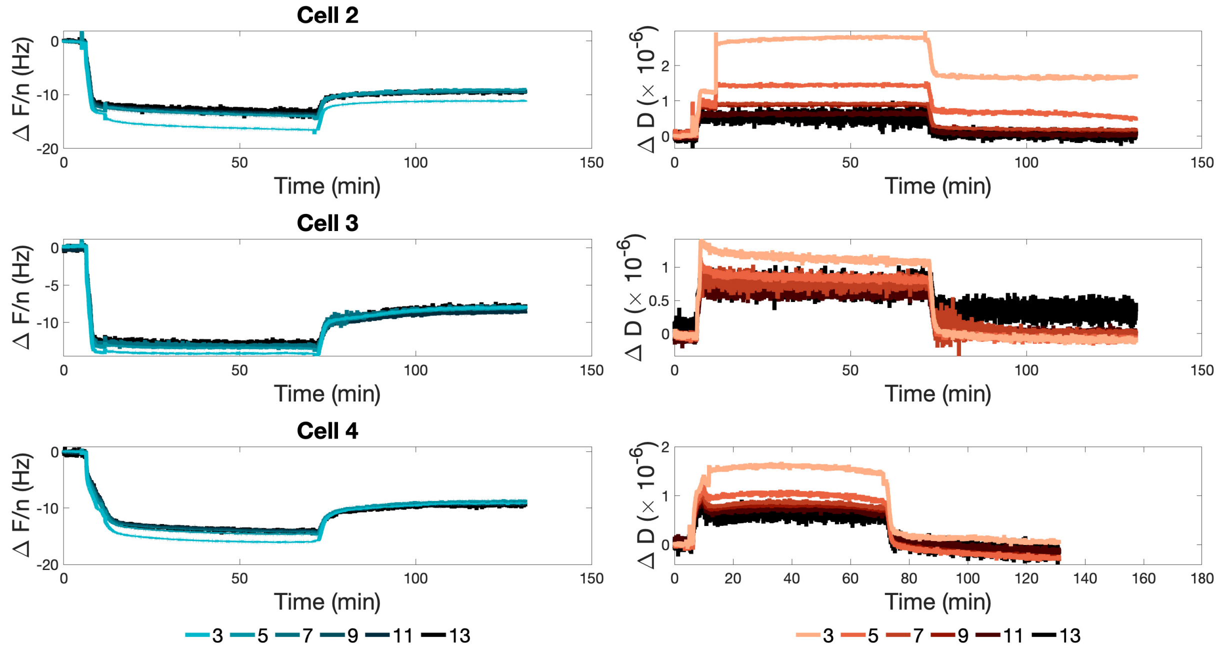

Figure S13: Quartz-crystal microbalance with dissipation monitoring data after injection of [14]Hst5 in 10 mM NaCl buffer. All overtones for each measurement cell are presented.

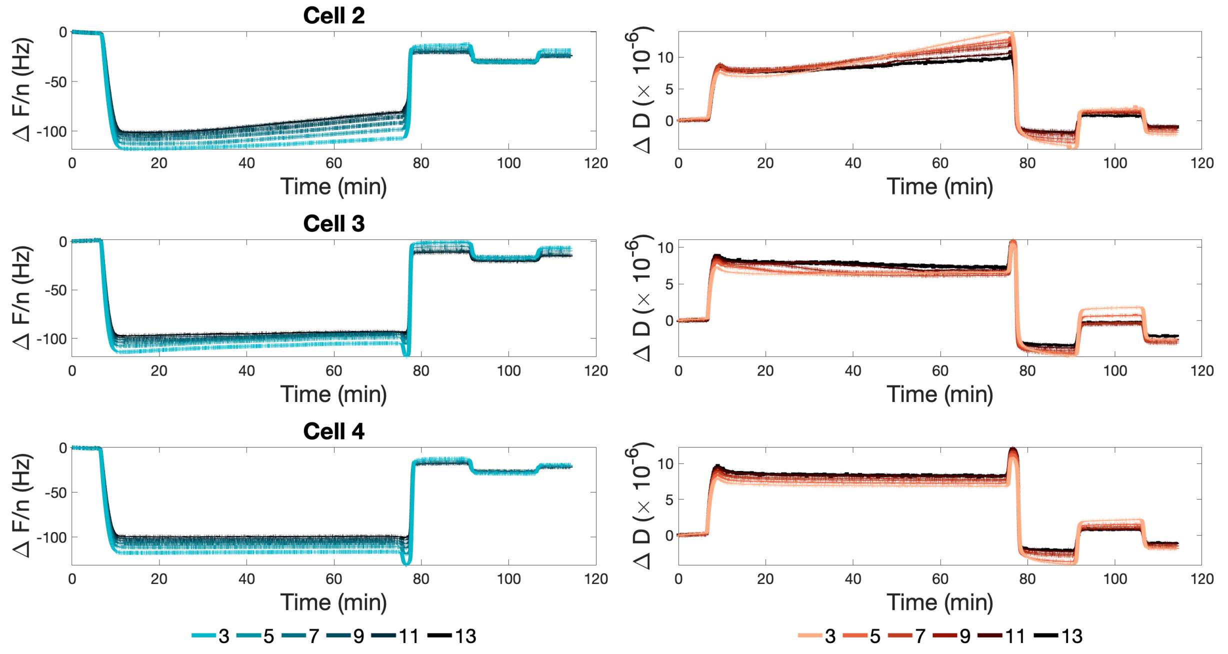

Figure S14: Quartz-crystal microbalance with dissipation monitoring data of the supported lipid bilayer formation to which [14]Hst5 in 150 mM NaCl buffer were injected to. All overtones for each measurement cell are presented.

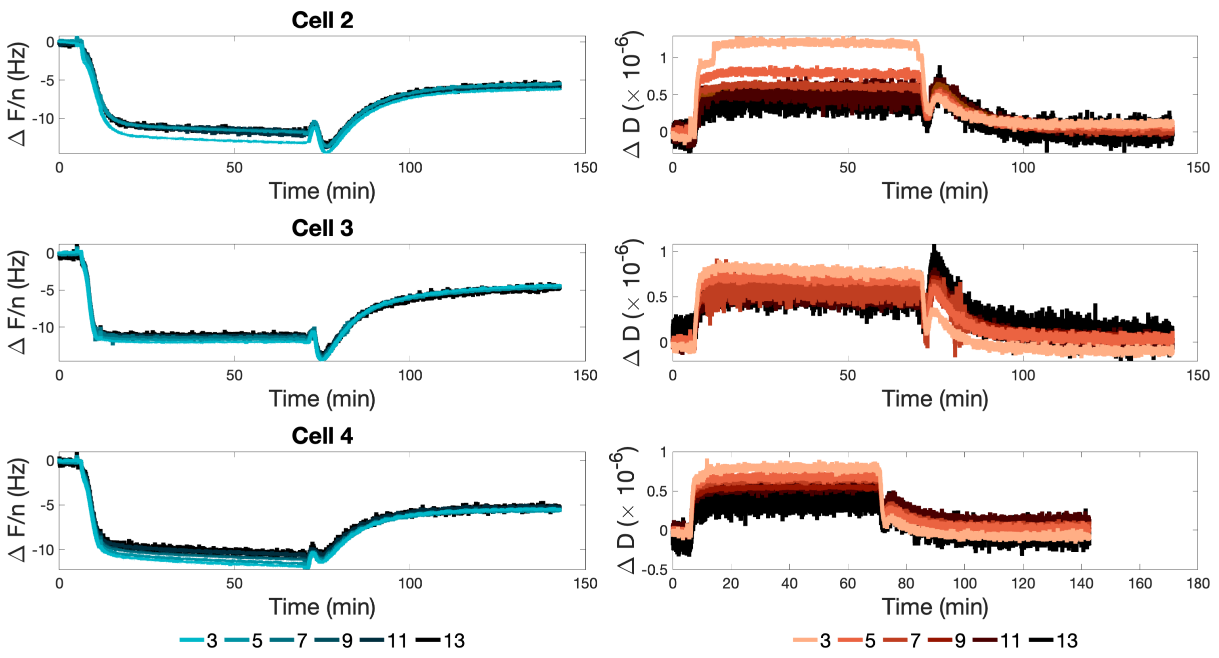

Figure S15: Quartz-crystal microbalance with dissipation monitoring data after injection of ^{14}Hst5 in 150 mM NaCl buffer. All overtones for each measurement cell are presented.

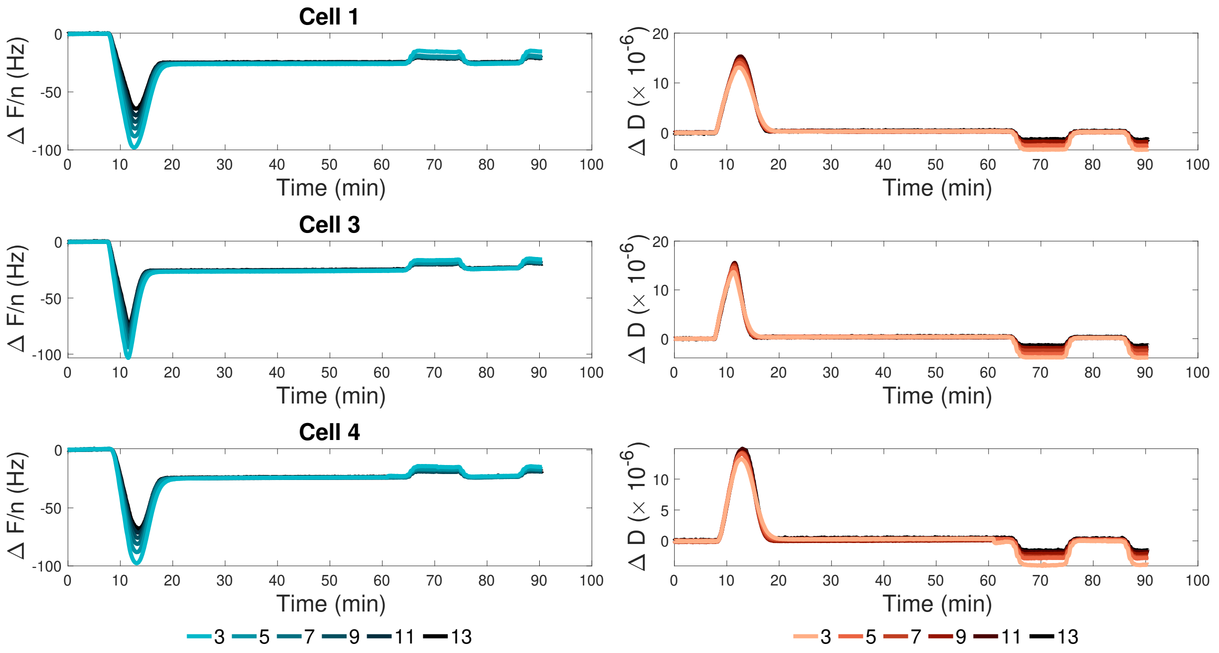

Figure S16: Quartz-crystal microbalance with dissipation monitoring data of the supported lipid bilayer formation to which ^{24}Hst5 in 10 mM NaCl buffer were injected to. All overtones for each measurement cell are presented.

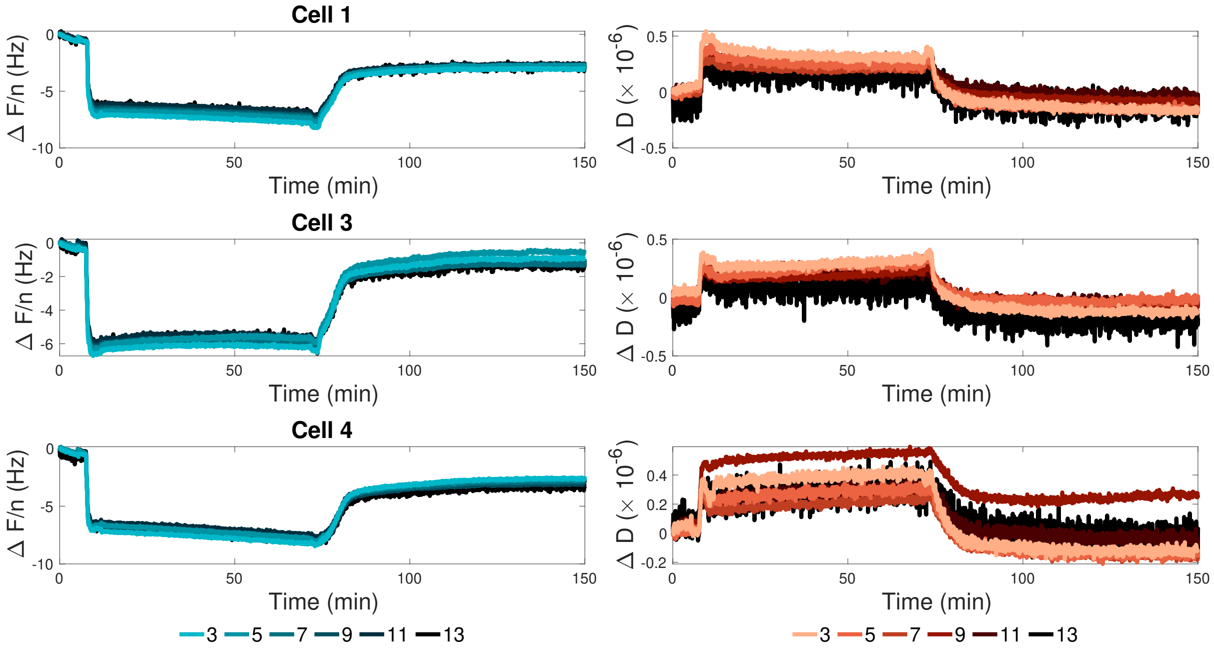

Figure S17: Quartz-crystal microbalance with dissipation monitoring data after injection of ^{24}Hst5 in 10 mM NaCl buffer. All overtones for each measurement cell are presented.

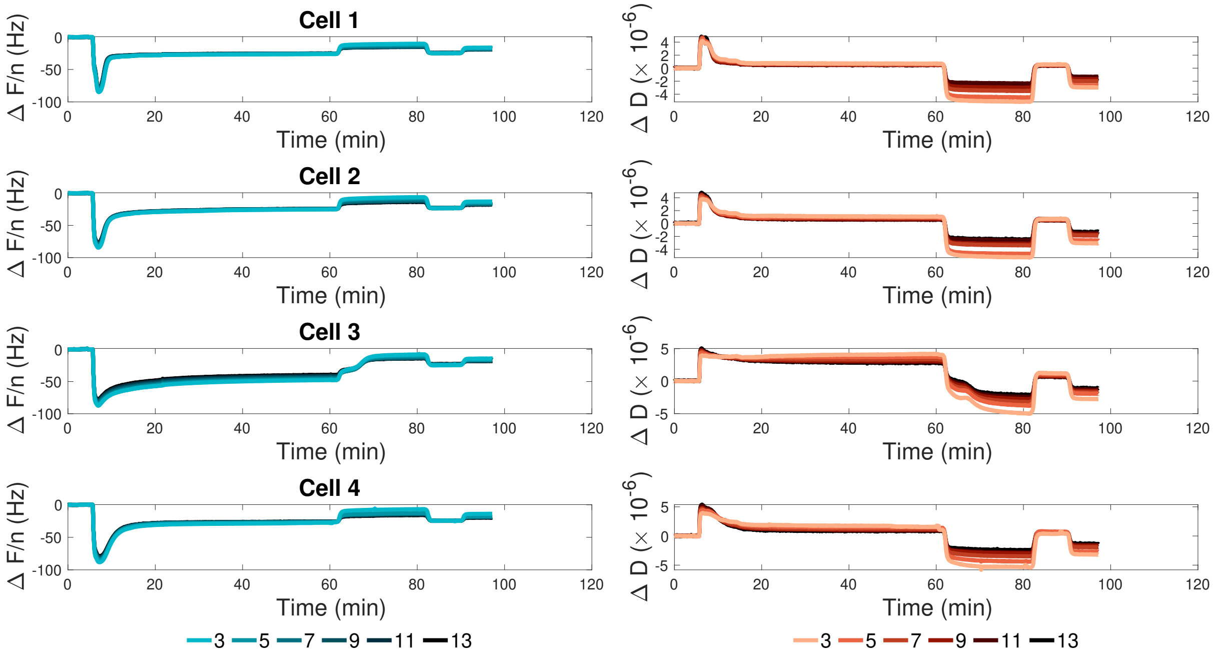

Figure S18: Quartz-crystal microbalance with dissipation monitoring data of the supported lipid bilayer formation to which ^{24}Hst5 in 150 mM NaCl buffer were injected to. All overtones for each measurement cell are presented.

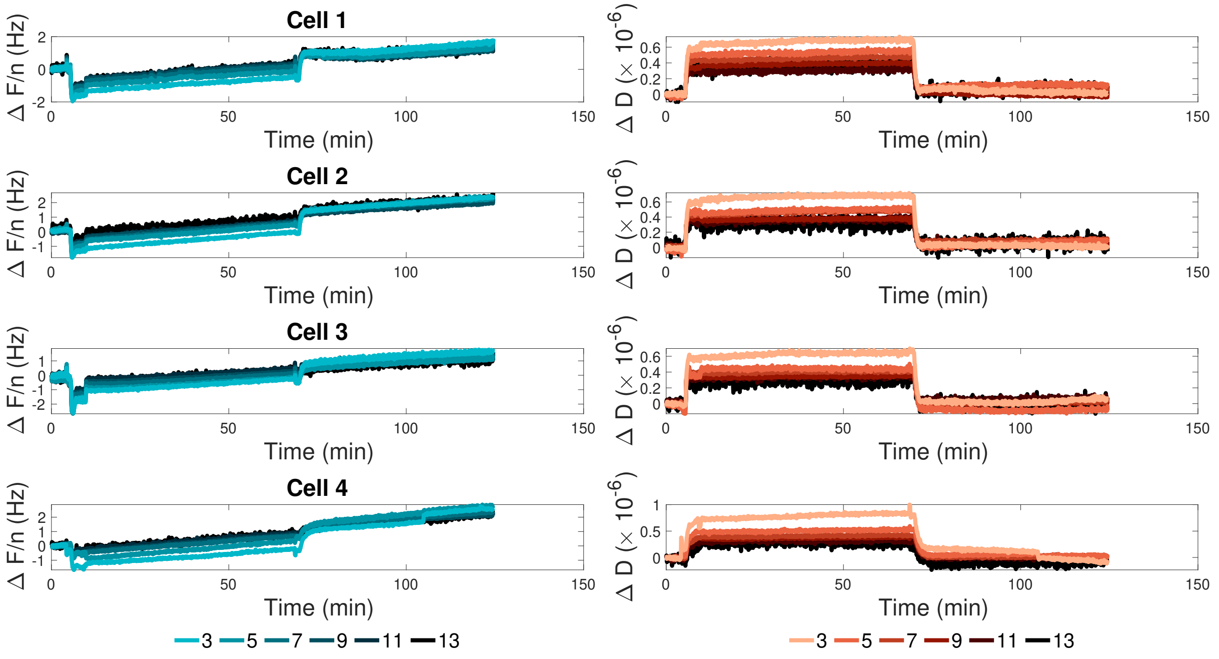

Figure S19: Quartz-crystal microbalance with dissipation monitoring data after injection of ^{24}Hst5 in 150 mM NaCl buffer. All overtones for each measurement cell are presented.

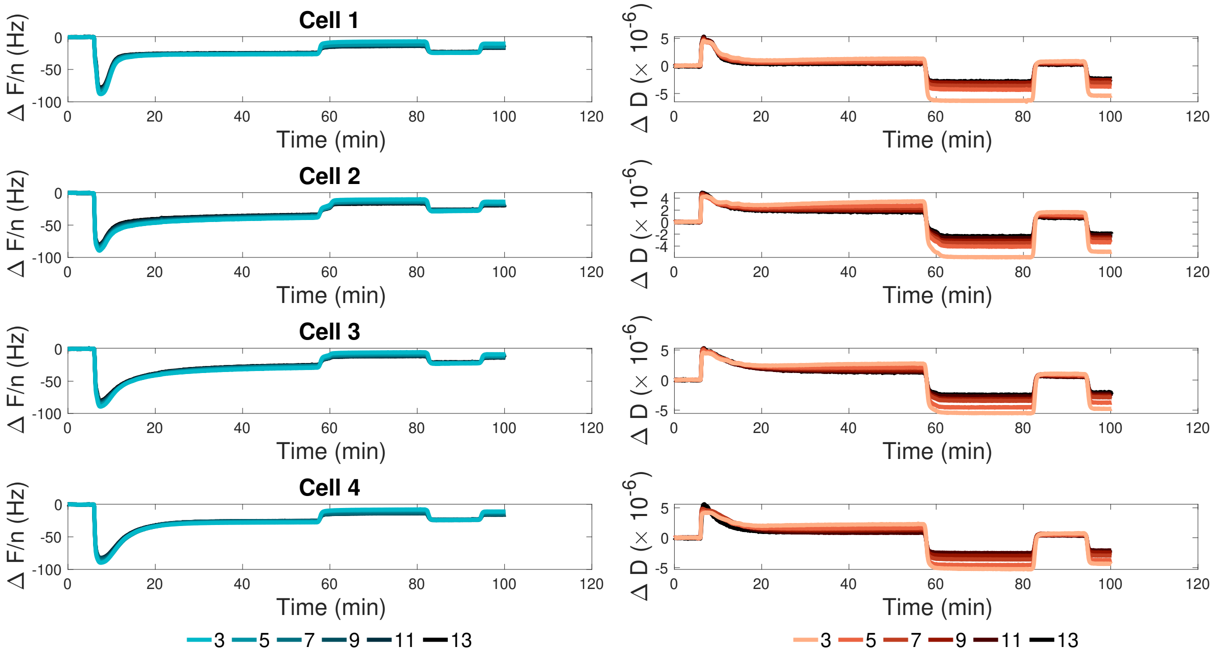

Figure S20: Quartz-crystal microbalance with dissipation monitoring data of the supported lipid bilayer formation to which ^{48}Hst5 in 10 mM NaCl buffer were injected to. All overtones for each measurement cell are presented.

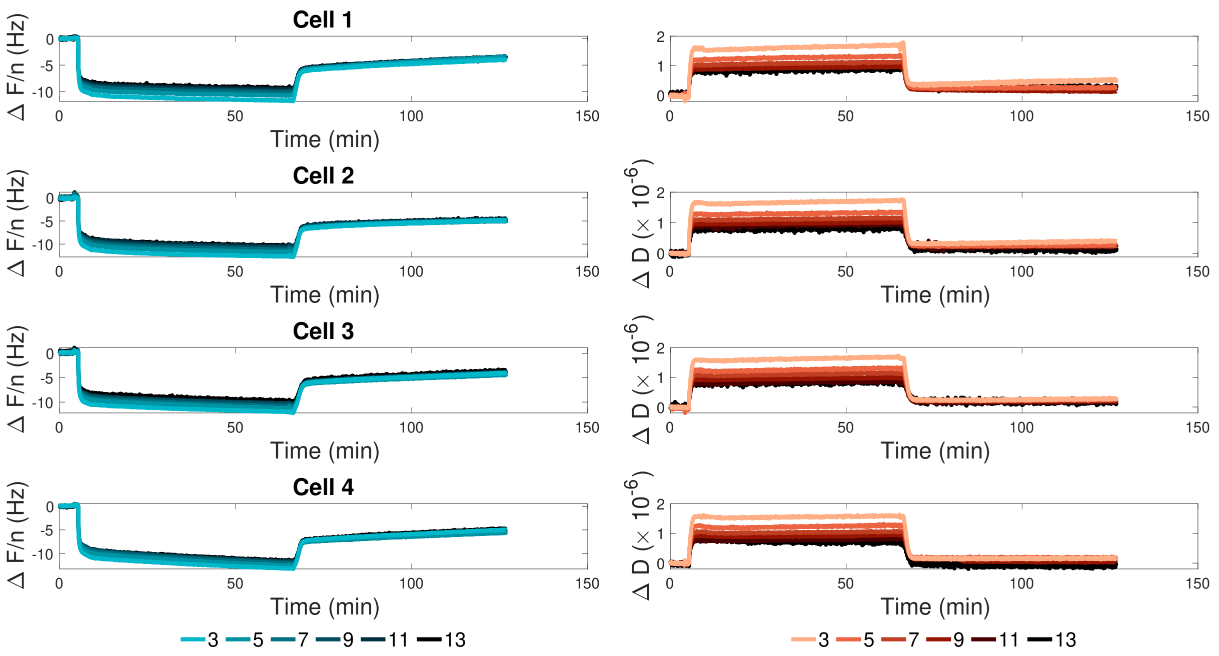

Figure S21: Quartz-crystal microbalance with dissipation monitoring data after injection of ^{48}Hst5 in 10 mM NaCl buffer. All overtones for each measurement cell are presented.

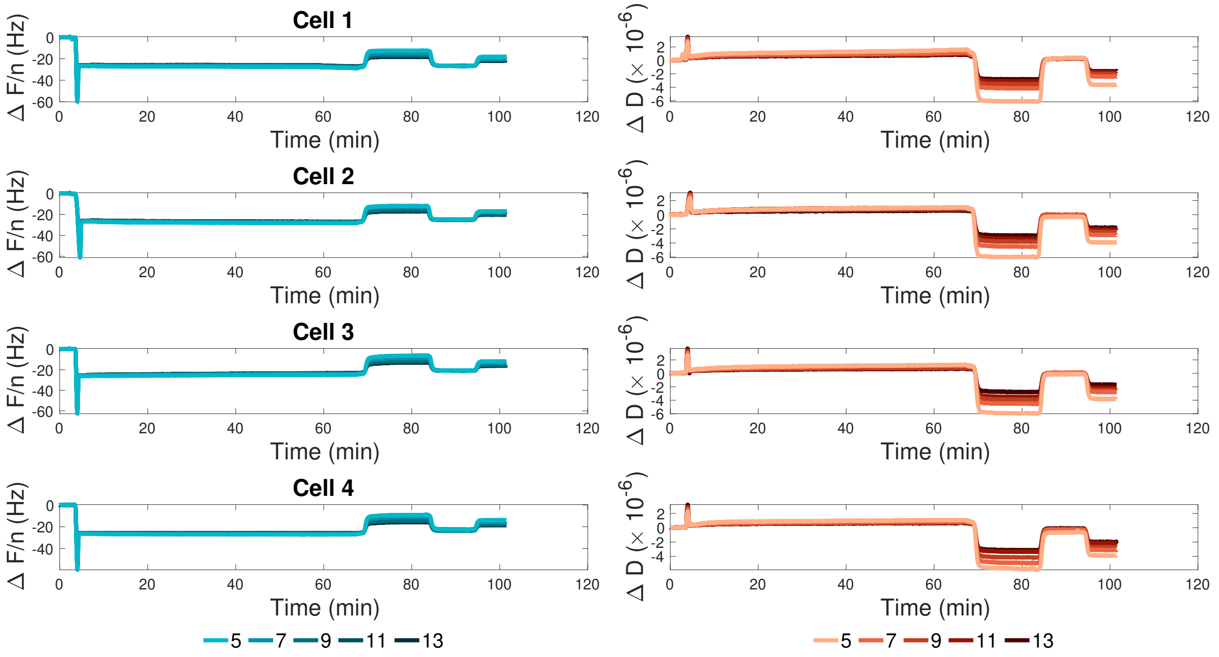

Figure S22: Quartz-crystal microbalance with dissipation monitoring data of the supported lipid bilayer formation to which ^{48}Hst5 in 150 mM NaCl buffer were injected to. All overtones for each measurement cell are presented, except F3.

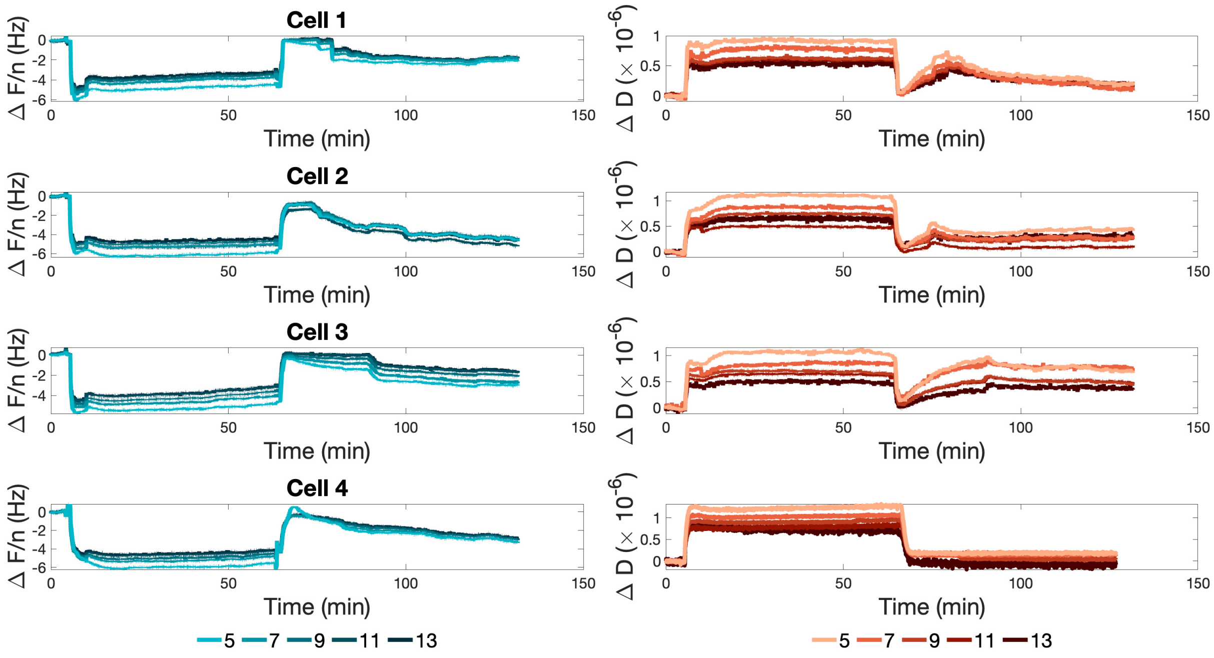

Figure S23: Quartz-crystal microbalance with dissipation monitoring data after injection of ^{48}Hst5 in 150 mM NaCl buffer. All overtones for each measurement cell are presented, except F3.

S5 Computer Simulations

Table S3: Radius of gyration (R_g), and end-to-end distance (R_{ee}) obtained from simulated peptides in both 10 and 150 mM NaCl.

Peptide	R_g [Å]		R_{ee} [Å]	
	10 mM	150 mM	10 mM	150 mM
^{14}Hst5	9.87 ± 0.020	9.96 ± 0.022	23.53 ± 0.076	24.30 ± 0.087
^{24}Hst5	13.96 ± 0.036	13.73 ± 0.034	33.92 ± 0.11	33.79 ± 0.13
^{48}Hst5	22.72 ± 0.062	20.97 ± 0.058	57.08 ± 0.19	52.48 ± 0.18

Table S4: Radius of gyration, R_g, obtained from the different peptides at different surface distances obtained from the simulations.

Peptide	Rg [Å]	
	10 mM	150 mM
**20 Å**		
^{14}Hst5	9.3 ± 0.1	9.3 ± 0.0
^{24}Hst5	12.9 ± 0.1	12.8 ± 0.1
^{48}Hst5	20.0 ± 0.4	20.3 ± 0.3
**40 Å**		
^{14}Hst5	10.0 ± 0.0	9.8 ± 0.0
^{24}Hst5	13.0 ± 0.1	12.9 ± 0.0
^{48}Hst5	19.4 ± 0.2	18.9 ± 0.1
**100 Å**		
^{14}Hst5	10.2 ± 0.0	10.0 ± 0.0
^{24}Hst5	13.4 ± 0.1	13.4 ± 0.0
^{48}Hst5	20.8 ± 0.1	20.1 ± 0.1

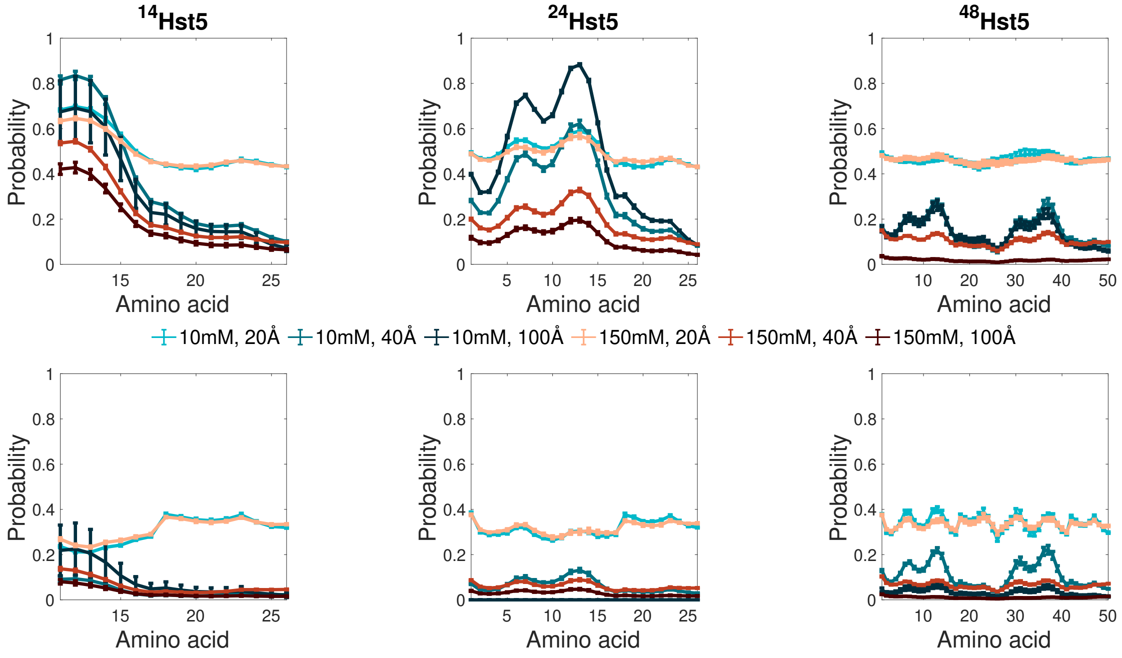

Figure S24: Adsorption profile of the three different peptides to a surface mimicking a bilayer (top) with a total charge of -78e (-0.5e/point) and a surface mimicking a silica surface (bottom) with a total charge of -49.5e (-0.05e/point) at 10 and 150 mM NaCl.

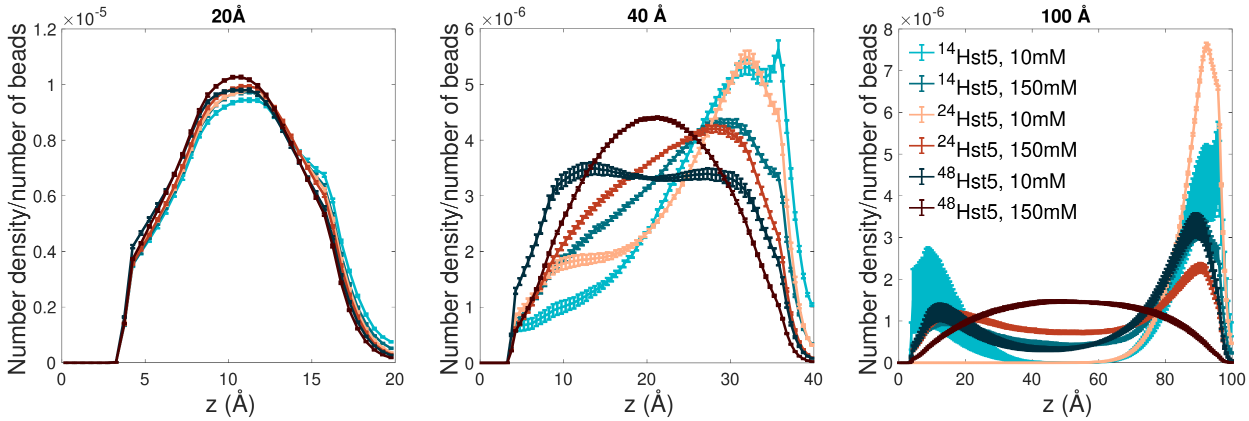

Figure S25: Number density summed over all the beads in a chain in z-direction divided by the number of beads in each chain. The surfaces are located at each end of the z-direction, where the surface mimicking the silica surface is placed at $z = 0$, and the one mimicking the bilayer is on the other end. Results from two different screening lengths are presented, corresponding to an ionic strength of 10, and 150 mM NaCl, respectively. Points were obtained every 0.5 Å. The depleted region close to the silica surface is due to hard-sphere repulsion. This is not observed for the bilayer as that surface is not as densely packed with particles. Hence, the chain is allowed in between the surface particles.

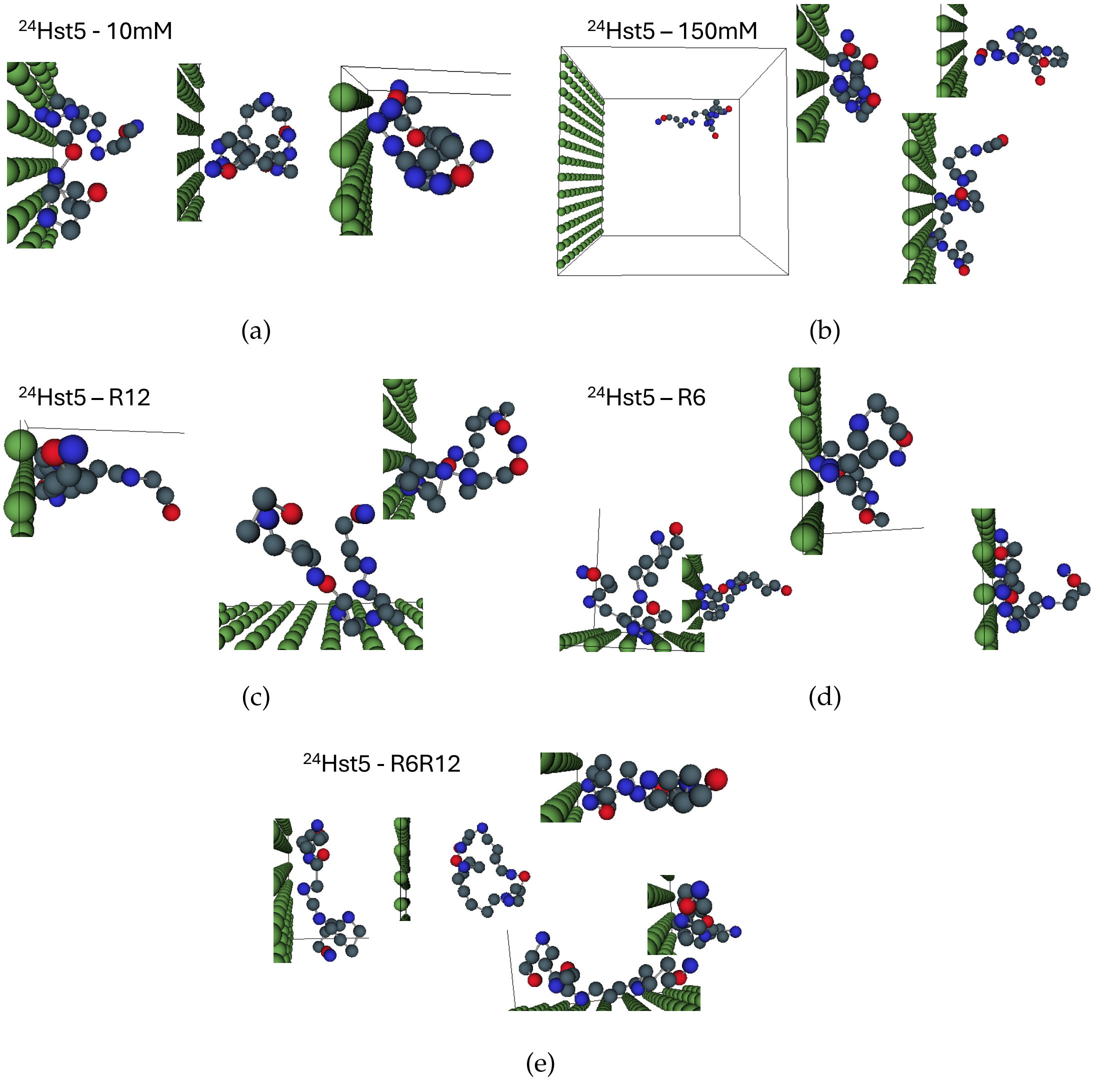

Figure S26: Snapshots obtained for ^{24}Hst5 where the charge of different arginines have been set to 0. The green spheres represent the surface, positively charged amino acids are presented in blue, negatively charged ones are in red, and uncharged amino acids are represented by grey spheres. Counter ions have been removed for clarity.

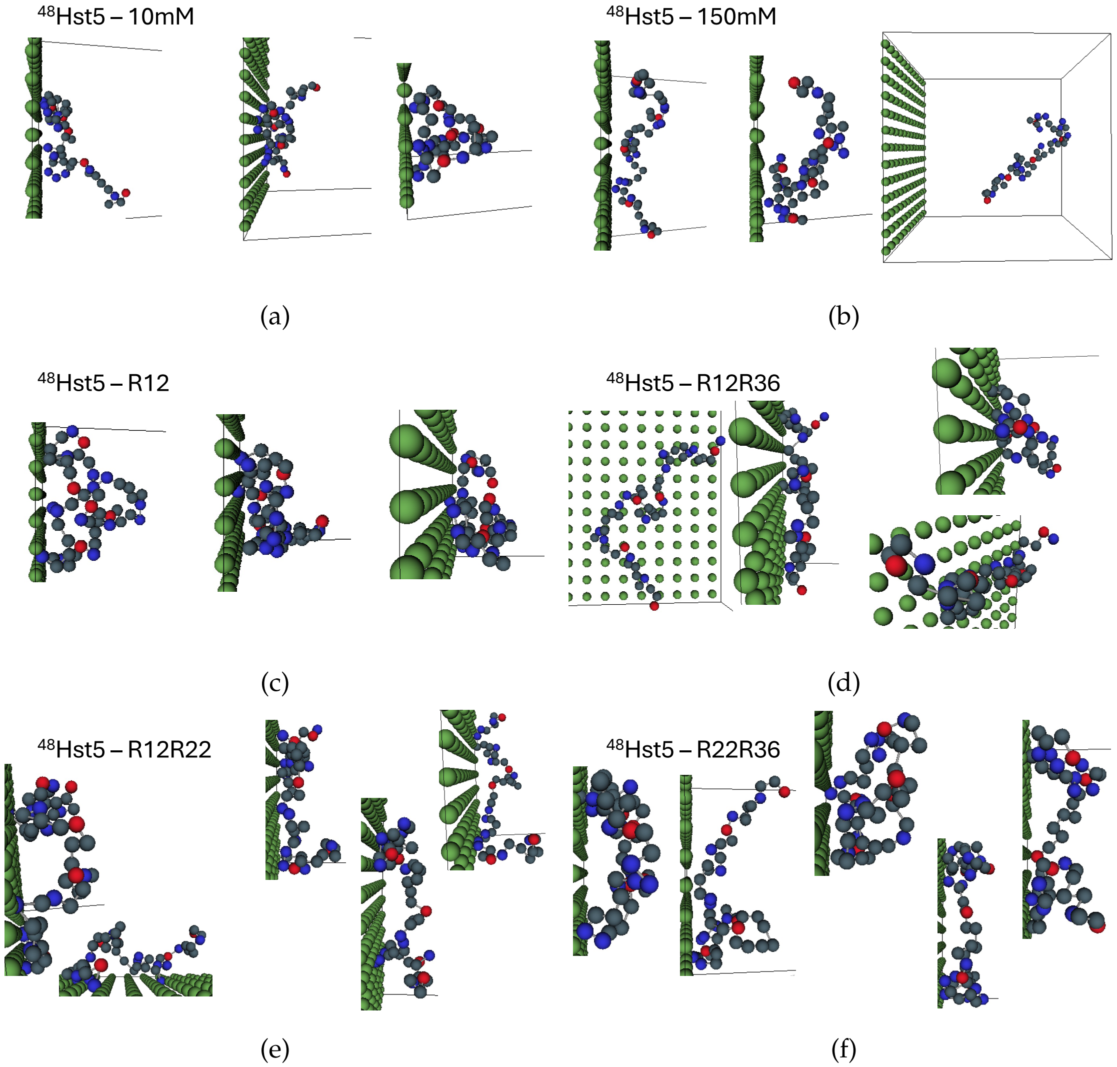

Figure S27: Snapshots obtained for ^{48}Hst5 where the charge of different arginines have been set to 0. The green spheres represent the surface, positively charged amino acids are presented in blue, negatively charged ones are in red, and uncharged amino acids are represented by grey spheres. Counter ions have been removed for clarity.

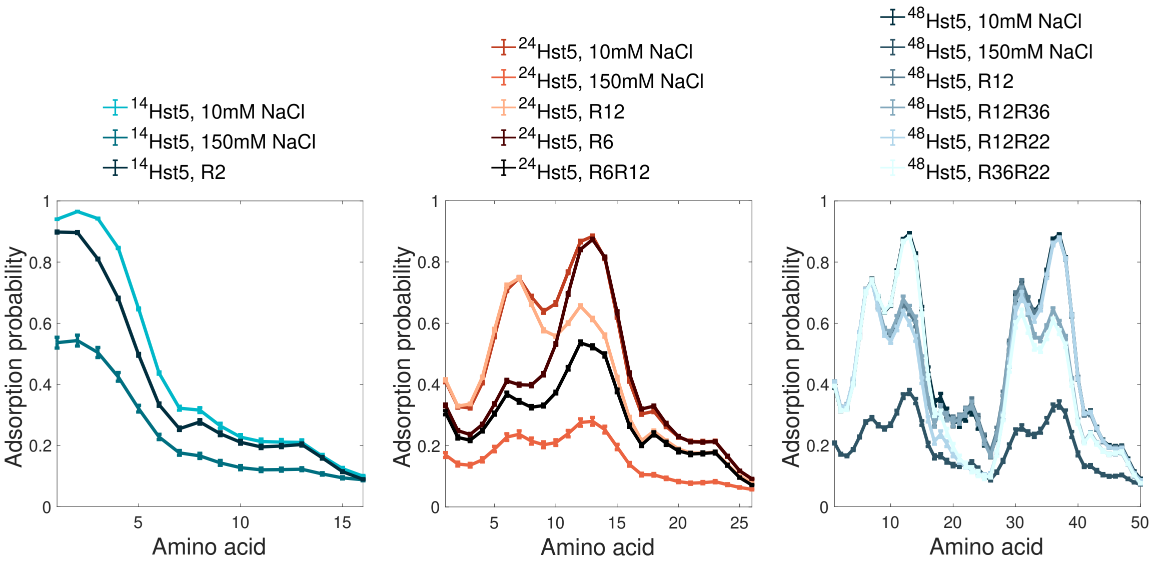

Figure S28: Simulated adsorption probabilities obtained for ^{14}Hst5 (left), ^{24}Hst5 (middle), and ^{48}Hst5 (right) to the surface in different salt concentrations, as well as different mutations made on the peptide.

S6 Nuclear Localization Signal Predictors

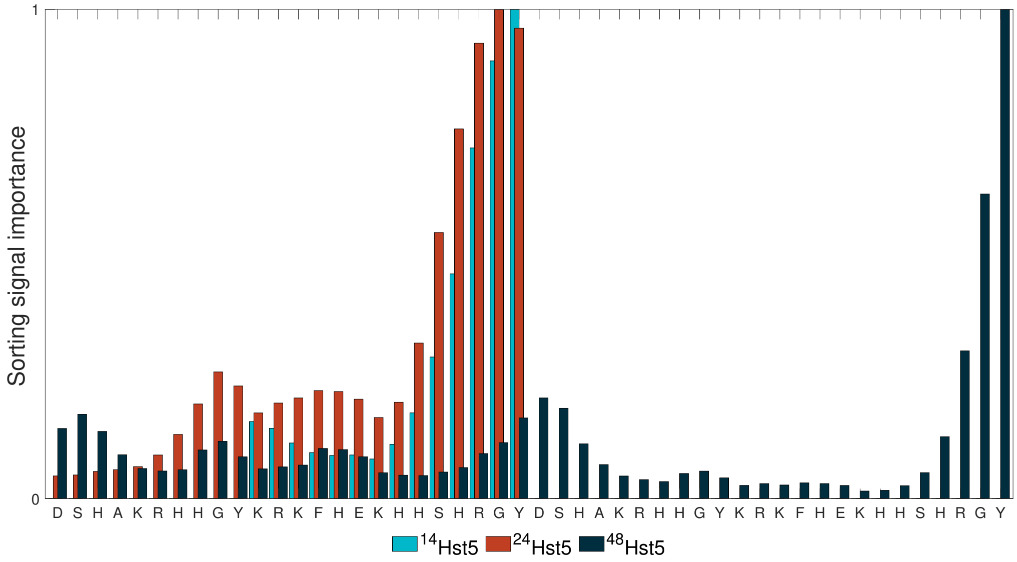

Figure S29: Importance per amino acid contributing to nuclear localization sequence predicted using DeepLoc-2.0.
